# Guided construction of single cell reference for human and mouse lung

**DOI:** 10.1038/s41467-023-40173-5

**Published:** 2023-07-29

**Authors:** Minzhe Guo, Michael P. Morley, Cheng Jiang, Yixin Wu, Guangyuan Li, Yina Du, Shuyang Zhao, Andrew Wagner, Adnan Cihan Cakar, Michal Kouril, Kang Jin, Nathan Gaddis, Joseph A. Kitzmiller, Kathleen Stewart, Maria C. Basil, Susan M. Lin, Yun Ying, Apoorva Babu, Kathryn A. Wikenheiser-Brokamp, Kyu Shik Mun, Anjaparavanda P. Naren, Geremy Clair, Joshua N. Adkins, Gloria S. Pryhuber, Ravi S. Misra, Bruce J. Aronow, Timothy L. Tickle, Nathan Salomonis, Xin Sun, Edward E. Morrisey, Jeffrey A. Whitsett, NHLBI LungMAP Consortium, Sara Lin, Yan Xu

**Affiliations:** 1 The Perinatal Institute and Section of Neonatology, Perinatal and Pulmonary Biology, Cincinnati Children’s Hospital Medical Center, 3333 Burnet Avenue, Cincinnati, OH 45229 USA; 2 Department of Pediatrics, University of Cincinnati College of Medicine, 3230 Eden Avenue, Cincinnati, OH 45267 USA; 3 Department of Medicine, University of Pennsylvania, Philadelphia, PA 19104 USA; 4 Penn-CHOP Lung Biology Institute, University of Pennsylvania, Philadelphia, PA 19104 USA; 5 Department of Cell and Developmental Biology, University of Pennsylvania, Philadelphia, PA 19104 USA; 6 Division of Biomedical Informatics, Cincinnati Children’s Hospital Medical Center, 3333 Burnet Avenue, Cincinnati, OH 45229 USA; 7 RTI International, Durham, NC 27709 USA; 8 Division of Pathology and Laboratory Medicine, Cincinnati Children’s Hospital Medical Center, 3333 Burnet Avenue, Cincinnati, OH 45229 USA; 9 Department of Pathology & Laboratory Medicine, University of Cincinnati College of Medicine, 3230 Eden Avenue, Cincinnati, OH 45267 USA; 10 Division of Pulmonary and Critical Care Medicine, Department of Medicine, Cedars-Sinai Medical Center, Los Angeles, CA 90048 USA; 11 Board of Governors Regenerative Medicine Institute, Cedars-Sinai Medical Center, Los Angeles, CA 90048 USA; 12 Biological Sciences Division, Pacific Northwest National Laboratory, Richland, WA 99352 USA; 13 Department of Pediatrics Division of Neonatology, University of Rochester Medical Center, Rochester, NY 14642 USA; 14 Data Sciences Platform, The Broad Institute, Cambridge, MA 02142 USA; 15 Department of Pediatrics, University of California at San Diego, 9500 Gilman Dr., La Jolla, CA 92093 USA; 16 Department of Biological Sciences, University of California at San Diego, 9500 Gilman Dr, La Jolla, CA 92093 USA; 17 National Heart, Lung, and Blood Institute, National Institutes of Health, Bethesda, MD 20892 USA

**Keywords:** Data integration, RNA sequencing, Software

## Abstract

Accurate cell type identification is a key and rate-limiting step in single-cell data analysis. Single-cell references with comprehensive cell types, reproducible and functionally validated cell identities, and common nomenclatures are much needed by the research community for automated cell type annotation, data integration, and data sharing. Here, we develop a computational pipeline utilizing the LungMAP CellCards as a dictionary to consolidate single-cell transcriptomic datasets of 104 human lungs and 17 mouse lung samples to construct LungMAP single-cell reference (CellRef) for both normal human and mouse lungs. CellRefs define 48 human and 40 mouse lung cell types catalogued from diverse anatomic locations and developmental time points. We demonstrate the accuracy and stability of LungMAP CellRefs and their utility for automated cell type annotation of both normal and diseased lungs using multiple independent methods and testing data. We develop user-friendly web interfaces for easy access and maximal utilization of the LungMAP CellRefs.

## Abstract (web summary)

Accurate cell-type identification is vital for single-cell analysis. Here, the authors develop a computational pipeline called “LungMAP CellRef” for efficient, automated cell-type annotation of normal and disease human and mouse lung single-cell datasets.

## Introduction

Single-cell RNA-seq (scRNA-seq) analysis is being widely applied in biomedical research, enabling the study of complex organs, such as the lung, at unprecedented scale and resolution, and transforming our understanding of organ development and disease^1–4^. Accurate cell type identification is a necessary step in single-cell data analysis that usually requires time-consuming processes to optimize computational parameters followed by manual inspection that requires domain expertise. With the increasing number of published scRNA-seq datasets and the release of large-scale cell atlases, advanced computational tools^5–7^ have been developed using annotated datasets to predict cell identities in new datasets. Common issues related with the use of a published scRNA-seq dataset as a reference for supervised classification of user-supplied datasets include the lack of comprehension (missing cell types), inclusion of speculative cell types/states that have not been functionally validated, technology specific-biases in the reference or query, and insufficient power to represent the repertoire of common healthy lung cell types. The lack of common cell type nomenclatures and guidelines for single cell transcriptomic studies also creates substantial technical challenges for data integration and comparison. Therefore, single-cell references with comprehensive cell types, functionally validated cell identities, and standardized nomenclature are much needed by the research community to optimize automated cell type annotation and facilitate data integration, sharing, and collaboration.

A growing number of community-wide efforts have been devoted to the development of common cell type nomenclature, including cell type ontologies of the Human Cell Atlas^8^ and mammalian brain^9^. Recently, the LungMAP consortium produced a LungMAP CellCards^10^, a rigorous catalog of lung cells based on a community-wide effort that synthesizes current functional and single-cell data from human and mouse lungs into a comprehensive and practical cellular census of lung cells. The current version of LungMAP CellCards catalogs major lung cell types and numerous immune cell subtypes, spanning the cellular heterogeneity present in diverse regions of normal lung, including trachea, bronchi, submucosal glands (SMG), and lung parenchyma^10^. These common cell type nomenclature efforts provide a scaffold and guideline for the ongoing development of a comprehensive lung single-cell reference for single-cell genomics analysis. In addition to curation, computational methods are further needed to utilize carefully curated literature knowledge as guidelines to accurately identify cell types using integrated single-cell datasets.

Here, we present a guided approach for cell atlas construction that directs the identification of lung reference cell populations according to a dictionary of pre-compiled cell type terms and molecular markers derived from CellCards. The pipeline consists of two key steps, first identifying a seed population for each cell type which best represents the cell identity in the dictionary, then mapping all cells to the seeds based on transcriptomic similarity to construct a complete single-cell reference, termed CellRef. Using this approach, we constructed and released a CellRef consisting of a total of 48 normal human lung cell types, which we named LungMAP Human Lung CellRef. Using the same approach, we identified seed cells for 40 mouse lung cell types and constructed the LungMAP Mouse Lung Development CellRef. We deployed this resource as multiple user-friendly web interfaces to facilitate easy access and maximize use of the LungMAP CellRefs. These interfaces include the use of the recently developed Azimuth interface^5^, which enables research investigators to annotate their own scRNA-seq dataset automatically using the LungMAP CellRefs, via automated supervised classification, prior user-annotation comparison, and exploration against the CellRef for any scRNA-seq input dataset. We developed functions to facilitate evaluation of automated cell type annotation results using CellRef marker genes. Using multiple independent methods and testing data, and benchmarking across different lung atlases, we demonstrate the accuracy and stability of LungMAP CellRefs and their utility for automated cell type annotation of both normal and diseased lungs. The present guided approach is implemented in R and is applicable for CellRef construction for other organs.

## Results

### Data collection and guided construction of a LungMAP single-cell reference

**Fig. 1:**
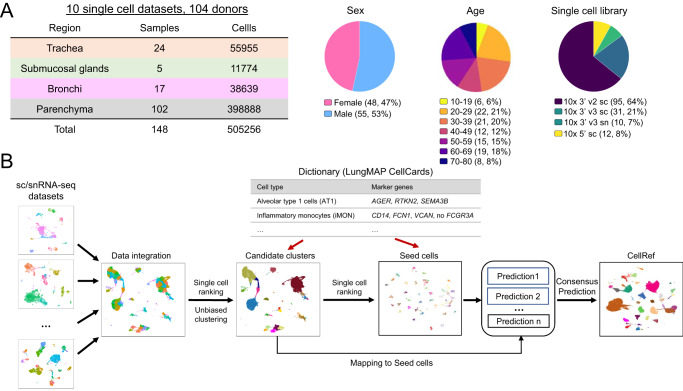
Data collection and the guided single-cell reference (CellRef) construction pipeline. **A** Characteristics of the collection of single cell/nucleus (sc/sn) RNA-seq datasets from normal human lung samples. **B** Schematic workflow for the LungMAP CellRef construction guided by using LungMAP CellCards as a cell type dictionary.

The LungMAP CellCards catalogued major lung cell types and their associated marker genes in multiple regions of normal lung, including trachea, bronchi, SMG, and lung parenchyma^10^. To construct a LungMAP human lung CellRef in accordance with the CellCards, we collected 10 large-scale sc/snRNA-seq datasets (8 published and 2 unpublished) from the four regions of human lung (Fig. 1A): Habermann et al.^11^ (*n* = 10 donors; parenchyma), Reyfman et al.^12^ (*n* = 8 donors; parenchyma), Adams et al.^13^ (*n* = 28 donors; parenchyma), Deprez et al.^14^ (*n* = 9 donors; trachea/bronchi/parenchyma), Travaglini et al.^15^ (*n* = 3 donors; bronchi/parenchyma), Goldfarbmuren et al.^16^ (*n* = 15 donors; trachea), Wang et al. (*n* = 3, small airway, neonatal/early pediatric samples excluded), Melms et al.^3^ (*n* = 7, parenchyma), CCHMC LungMAP cohort (*n* = 5, bronchus SMG, unpublished) and UPenn LungMAP cohort (*n* = 16, parenchyma, unpublished). This collection contains data from similar numbers of female and male donors (*n* = 48 and 55, respectively; 1 unannotated) (Fig. 1A; Supplementary Data 1). The median age of donors was 41 years (interquartile range [IQR], 29−61 years; 1 unannotated). Data were generated from three 10x chromium single cell libraries: Single Cell 3’ sequencing kit based on v2/v3 and Single Cell 5’ chemistry. In total, 505,256 lung cells from 148 sc/snRNA-seq of normal human lung samples from 104 donors were used for LungMAP human lung CellRef construction (Supplementary Data 1).

The integration of such a large and complex single-cell data collection is challenging due to the huge batch differences associated with both biological (i.e., different donor and different anatomic regions) and technical variations (e.g., sample preparations by different protocols from different research institutions). To perform accurate single-cell reference construction, we developed a computational pipeline which combines batch correction, unsupervised cell clustering, single-cell ranking, power analysis, and automated cell type annotation to consolidate single-cell datasets and annotate cell identities guided by a pre-defined cell type dictionary (i.e., LungMAP CellCards) (Fig. 1B). We utilized both positive and negative markers to improve the sensitivity to distinguish cell types sharing similar gene expression patterns and marker genes, for example, lung goblet cells (*MUC5AC* + */MUC5B*+) and SMG mucous (*MUC5AC*-*/MUC5B*+). The pipeline consists of four major steps (Fig. 1B, “Methods”). First, we tried the mutual nearest neighbor (MNN) matching method in Monocle 3, Seurat’s reciprocal principal component analysis (RPCA) based integration^5^ and Harmony^17^ for batch correction. In addition, we applied a recently described cluster stability assessment framework to quantitatively assess and compare the cluster stability of different integration methods based on three independent statistical metrics to quantitively assess the data integration^18^. MNN outperforms RPCA and Harmony on both the UMAP inspection and cluster stability metrics measurement, we therefore set the mutual nearest neighbor (MNN) matching method in Monocle 3 as default, and Seurat’s reciprocal principal component analysis (RPCA) based integration and Harmony as alternatives (Supplementary Fig. 1). Next, seed identification was performed (steps 2 and 3 in Fig. 1B). This is a unique feature of our approach. We aim to identify a core set of cells that best match to the identity of each cell type in the CellCards dictionary. We perform unbiased clustering analysis and determine candidate cell clusters for each cell type based on the expression of marker genes in the dictionary. The use of unsupervised clustering in this step provides an opportunity to discover new cell types that are not yet defined in the dictionary. To identify the best seed cells, we developed a single-cell ranking method that first ranks cells based on expression of each cell specific marker gene in the CellCards dictionary and then aggregates the rankings of those markers for a given cell type to identify seed cells for the cell type. We performed a power analysis to determine the minimum number of seed cells required. The last step is consensus label transfer. We applied multiple machine learning methods (e.g., Seurat’s label transfer and SingleR) to map all other cells to the seed cells and determine their cell types based on the seed annotation. Cells that have consistent cell type predictions in all methods will be included in the CellRef. The last step can be repeated to include newly collected datasets into the CellRef by mapping them to the seed cells. We implemented this cell-type-dictionary guided CellRef construction pipeline in R and hosted its development and documentation in github: https://github.com/xu-lab/CellRef^19^.

### The LungMAP Human Lung CellRef

**Fig. 2:**
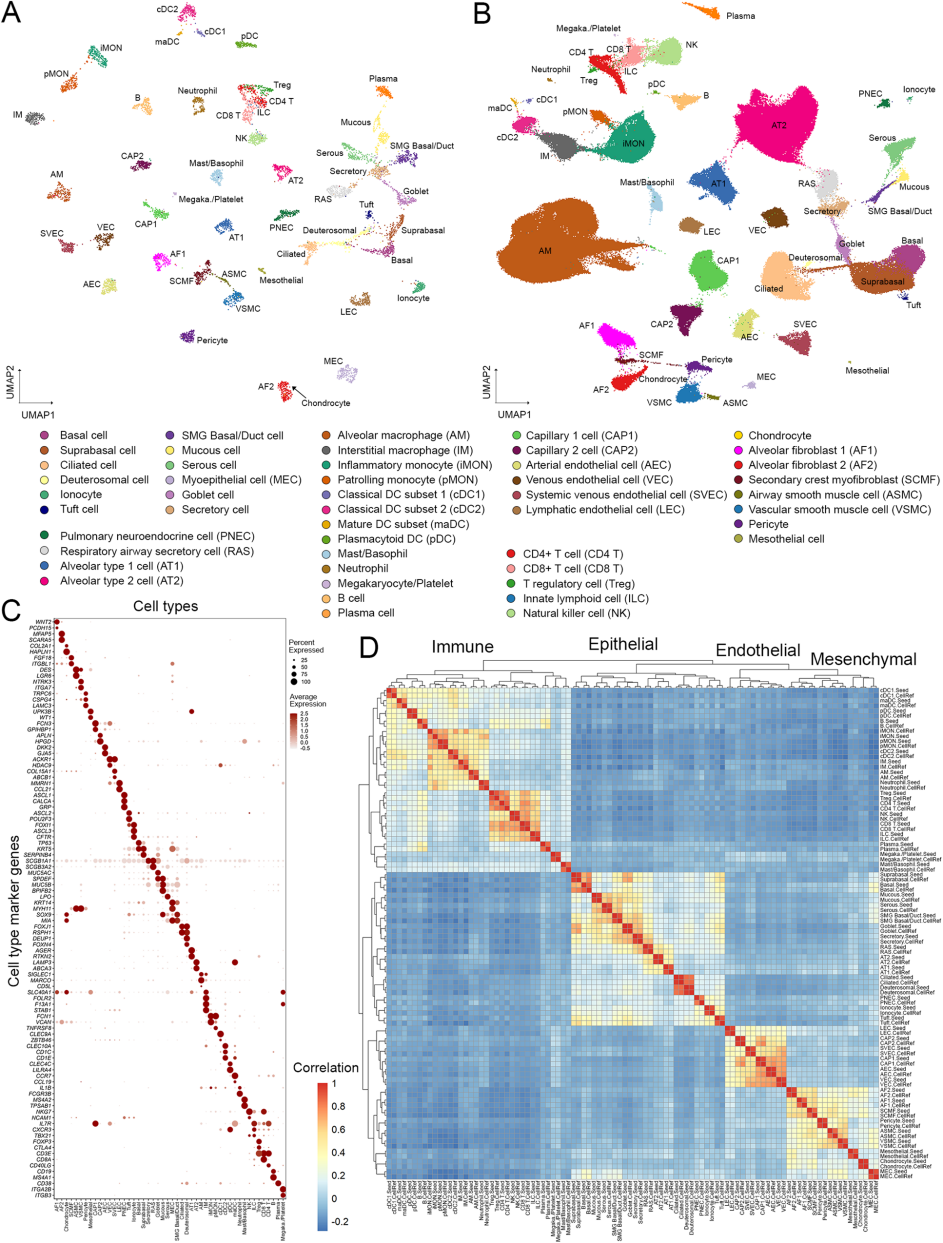
The construction of LungMAP Human Lung CellRef. **A** Uniform manifold approximation and projection (UMAP) visualization of seed cells representing 48 lung cell types of normal human lung, termed LungMAP Human Lung CellRef Seed. Cells were colored by their predicted seed identities. **B** UMAP visualization of the complete single-cell reference for normal human lung, denoted as LungMAP Human Lung CellRef, which contains 347,970 cells from 104 donors and defines 48 cell types in normal human lung. Cells were colored by their predicted identities. **C** Validation of the seed cell identity using the expression of cell type selective marker genes derived from LungMAP CellCards. **D** Reconstruction of cell lineage relationships using hierarchical clustering analysis of cell type pseudo-bulk gene expression profiles. Color represents Pearson’s correlation value of pseudo-bulk expression profiles. Labels ending with “.Seed” represent pseudo-bulk profiles created by averaging gene expression in the cells of each cell type in the human lung CellRef Seed, while labels ending with “.CellRef” represent pseudo-bulk profiles created using gene expression of each cell type in the complete human lung CellRef.

Using this guided approach and a cell type dictionary derived from LungMAP CellCards (Supplementary Data 2), we identified 8,080 seed cells representing 48 normal human lung cell types, termed LungMAP Human Lung CellRef Seed (Fig. 2A). Next, we mapped all other cells in our collection to the CellRef Seed cells and predicted cell type annotations using two independent methods, Seurat Label Transfer^5,20^ and SingleR^6^. Cells with consistent cell type annotations were combined with the CellRef Seed to form the LungMAP Human Lung CellRef (347,970 cells) (Fig. 2B, Supplementary Figs. 1–3, “Methods”).

The CellRef includes the following CellCards cells: 12 epithelial (AT1, AT2, basal, ciliated, goblet, myoepithelial [MEC], mucous, PNEC, secretory, serous, Tuft cells, and ionocytes); 5 endothelial (arterial, venous, lymphatic endothelial, capillary 1, and 2 cells), 8 mesenchymal (alveolar fibroblast 1 and 2 [AF1, AF2], airway and vascular smooth muscle cells [ASMC, VSMC], mesothelial cells, chondrocytes, pericytes, and myofibroblasts [SCMF]), and 16 immune cell types (alveolar and interstitial macrophage [AM, IM], inflammatory and patrolling monocytes [iMON, pMON], mast/basophils, neutrophils, B, plasma, NK, ILC, cDC1, cDC2, pDC, CD8 + T, CD4 + T, and T regulatory [Treg] cells). In addition to the known lung cell types, we extended the dictionary to incorporate 7 cell types that are not yet in the CellCards but have marker genes reported in recent scRNA-seq studies and are selectively expressed in our unbiasedly identified cell clusters, including deuterosomal cells^14^ (*DEUP1*, *FOXN4*, *CDC20B*), suprabasal cells^14^ (*SERPINB4*, *KRT19*, *NOTCH3*), systemic venous endothelial cells^21^ (SVEC; marker genes: *COL15A1*, *ABCB1*, *ACKR1*), mature dendritic cell subset (maDC; marker genes: *CCR7*, *CCL19*, *LAD1*), megakaryocyte/platelets^15, 22^ (*ITGA2B*, *ITGB3*), SMG duct cells (*MIA*, *ALDH1A3*, *RARRES1*), and respiratory airway secretory cells (RAS; marker genes: *SCGB3A2*, *KLK11*, *SOX4*). We combined SMG basal and SMG duct cells into one mixed type, SMG Basal/Duct cell, since their marker genes were co-expressed in the same cell cluster in our data integration. Similarly, we combined mast and basophil cells into a mixed Mast/Basophil cell type. These mixed cell types likely result from the lack of clear known markers or insufficient numbers of cells in the subtypes to distinguish the heterogeneity of the cluster in the current CellRef. We performed uniform manifold approximation and projection for dimension reduction (UMAP) analysis on the LungMAP Human Lung CellRef. All cells, from trachea to alveoli, were projected into a common UMAP space and showed clear separations by the predicted cell identities (Fig. 2B).

To evaluate cell identities in the human lung CellRef Seed, we preformed the following validation analyses. Cell type marker genes were found to be selectively expressed in their corresponding seed cells, the majority having high cell type specific expression frequencies, suggesting that the identities of the seed cells were consistent with the cell type dictionary (Fig. 2C). To further validate the identities of the seed cells, we created pseudo-bulk gene expression profiles for each cell type by averaging gene expression in its seed cells, measured their correlations, and performed hierarchical clustering analysis, demonstrating that cell types were first unbiasedly clustered by their major cell lineages and then by sub-lineages (Fig. 2D). The pseudo-bulk profile of SMG myoepithelial cells (MEC) co-clustered with mesenchymal cells and was positively correlated with both SMG Basal/Duct cells and smooth muscle cells, consistent with their complex cell nature. UMAP analysis showed that the seed cells formed dense cell clusters and clearly distinguished all cell types except closely related T cell subtypes (i.e., Treg and ILC are clustered with CD8/4 T cells), supporting distinct transcriptomic patterns of cell types in the CellRef Seed and a high similarity of the seed cells for each cell type (Fig. 2A). In summary, using our guided approach, we developed the LungMAP Human Lung CellRef Seed, a collection of seed cells for 48 normal lung cell types which can serve as a simplified version of CellRef with cell identities in accordance with a cell type dictionary derived from the LungMAP CellCards.

To validate the similarity of cell identities in the CellRef Seed and the full CellRef, we created pseudo-bulk profiles for the cell types in the CellRef, combined them with the pseudo-bulk profiles generated using the CellRef Seed, measured correlations among all pseudo-bulk profiles, and performed hierarchical clustering analysis. Like the CellRef Seed, the pseudo-bulk profiles of the cell types in the full CellRef were also first clustered by their major cell lineages and then by sub-lineages. Moreover, each of them was well correlated with the pseudo-bulk profile of the same cell type created using the CellRef Seed (Fig. 2D). Taken together, these results validated the identities of cell types in our constructed LungMAP Human Lung CellRef.

### The LungMAP Mouse Lung Development CellRef

**Fig. 3:**
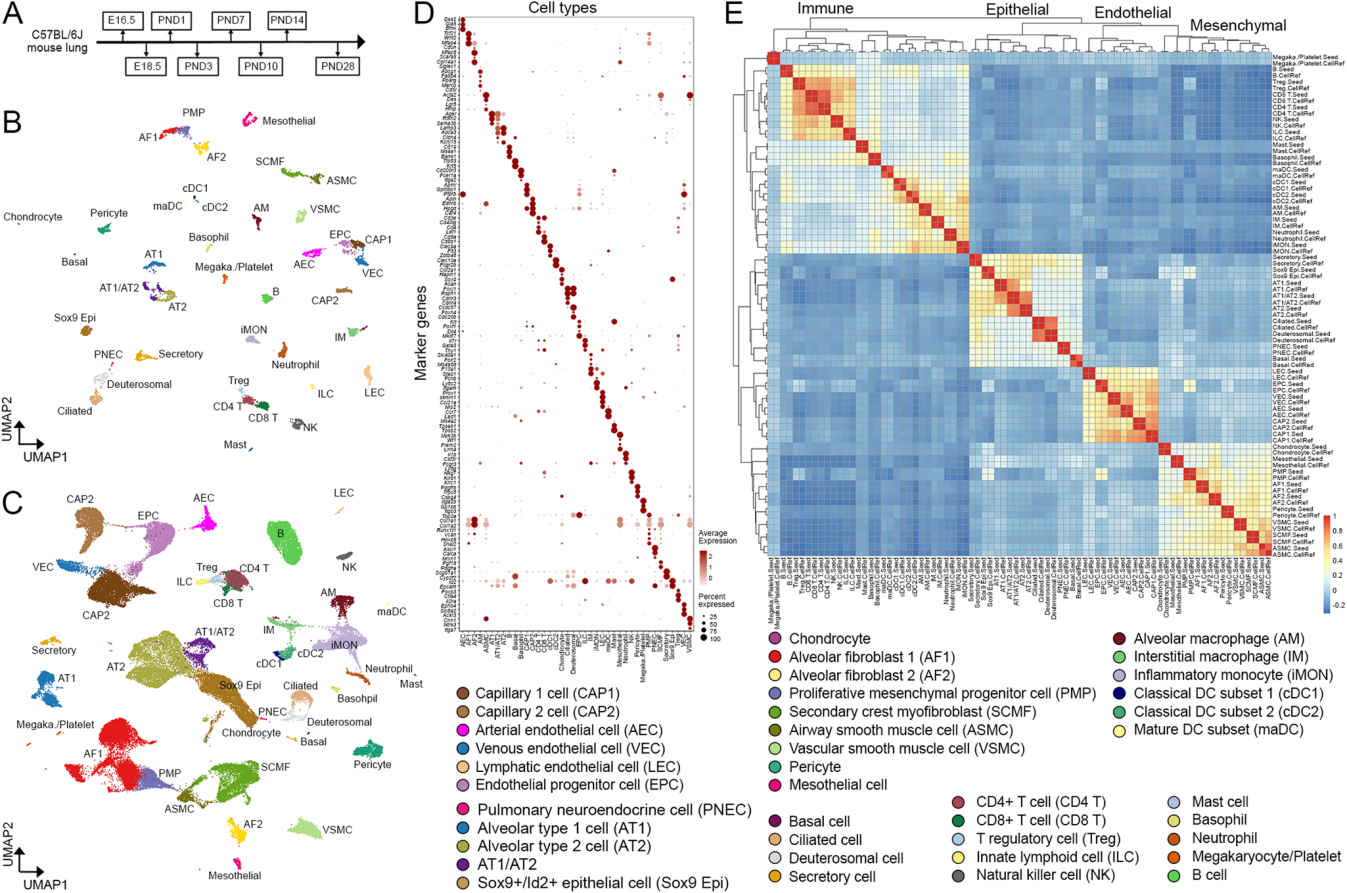
The construction of LungMAP Mouse Lung Development CellRef. **A** The developmental time points of mouse lung single-cell transcriptome data used for the guided CellRef construction. **B** Uniform manifold approximation and projection (UMAP) visualization of the seed cells representing 40 cell types of the developing mouse lung, termed LungMAP Mouse Lung Development CellRef Seed. Cells were colored by predicted seed identities. **C** UMAP visualization of CellRef for normal mouse lung development, named LungMAP Mouse Lung Development CellRef. Cells were colored by their predicted identities. **D** Validation of seed cell identities using expression of cell type selective marker genes. **E** Lineage relationships among mouse lung cell types were reconstructed using hierarchical clustering analysis using pseudo-bulk gene expression profiles. Color represents Pearson’s correlation value of pseudo-bulk expression profiles. Labels ending with “.Seed” represent pseudo-bulk profiles created by averaging gene expression in the cells of each cell type in the mouse lung CellRef Seed, while labels ending with “.CellRef” represent pseudo-bulk profiles created using gene expression of each cell type in the complete mouse lung CellRef.

Using the same approach, we constructed a cell type dictionary based on the LungMAP CellCards to define cell types in mouse lung during perinatal development, identified seed cells for each cell type (termed LungMAP Mouse Lung Development CellRef Seed”), and constructed a CellRef for mouse lung development (denoted as LungMAP Mouse Lung Development CellRef) using Drop-seq of mouse lungs (*n* = 95,658, 8 time points, 17 samples) from embryonic day 16.5 to postnatal day 28 (Fig. 3, Supplementary Fig. 4, Supplementary Data 3, 4). Because of the time course design, the mouse lung CellRef included more developmental progenitor cells and transitional cell states than the LungMAP Human Lung CellRef, including, *Sox9* + /*Id2*+ distal epithelial progenitor cell^23,24^, an AT1/AT2 cell population^22,25^ expressing both AT1 (*Ager*, *Hopx*) and AT2 (*Lamp3*, *Sftpc*, *Abca3*) cell markers in conjunction with *Cldn4*, *Krt19*, and *Krt8* (signature genes of recently reported PATS^26^, DATP^27^, or ADI^28^ cells), *Foxf1* + /*Kit*+ endothelial progenitor cells (EPC)^29^, and proliferative mesenchymal progenitor (PMP) cells^30,31^ (Fig. 3). In total, 40 mouse lung cell types have been identified with the guidance of the mouse lung cell type dictionary (Fig. 3, Supplementary Data 4). Cell identities were verified using expression of marker genes, UMAP visualization of cell types, pseudo-bulk expression and hierarchical clustering analysis-based cell lineage reconstruction, and cell type specific signature gene identification (Fig. 3). The construction of this LungMAP mouse lung CellRef in parallel with the human lung CellRef will enable cross comparisons for better understanding of how the cell types in mouse lung relate to the human lung and how data from mouse studies in the literature relate to human disease. The mouse CellRefs will continue to be expanded with adult time points and murine injury in the future.

### Interactive web-tools for search and display of the LungMAP CellRefs

**Fig. 4:**
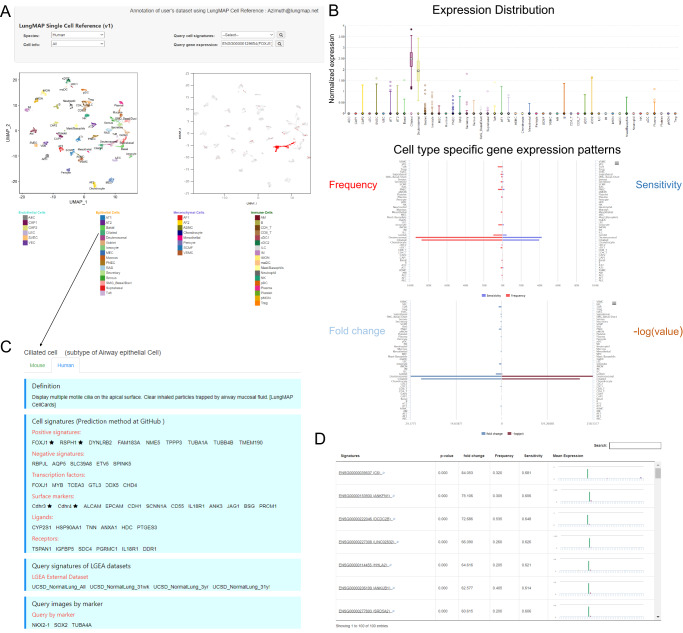
Online interactive exploration of LungMAP CellRef Seed using Lung Gene Expression Analysis (LGEA) web portal. The LungMAP Human Lung CellRef Seed was comprised of 8080 seed cells representing 48 normal lung cell types. **A** The “Gene Expression Query” interface allows users to input a gene of interest (top) and visualize of the expression of the queried gene in UMAP embeddings of cells (bottom), Colors represent the seed cell identities (bottom left) or the expression of the input gene (bottom right). **B** Visualization of the gene expression pattern (top: expression distribution; middle: expression frequency and sensitivity; bottom: fold change and *p*-value of differential expression) across all cell types in the CellRef Seed. Box center lines, bounds of the box, and whiskers indicate medians, first and third quartiles, and minimum and maximum values within 1.5×IQR (interquartile range) of the box limits, respectively. *P* value for each cell type was determined using a nonparametric binomial test^47^ for single-cell RNA-seq data by comparing the expression of *FOXJ1* in the cell type with its expression in all other cells in the CellRef Seed. See Fig. 4 source data table for number of cells in each cell type. **C** LGEA hosts comprehensive cell information related with the query cell type. **D** “Cell Signature Query” function retrieves signature gene expression statistics of a given cell type and bar-plot visualization of signature genes expression across all cell types in the CellRef Seed. *P* values were determined using a nonparametric binomial test^47^ for single-cell RNA-seq data by comparing gene expression in the ciliated cells (*n* = 200 cells) with all other cells (*n* = 7880 cells) in the CellRef Seed. In (**A**) and (**B**), *FOXJ1* expression was shown as example. In (**C**) and (**D**), Ciliated cells were used as example.

To facilitate data sharing and broad use of the resource, we developed several user-friendly web portals to host the LungMAP CellRefs online, including the LGEA LungMAP CellRef page for human and mouse (https://research.cchmc.org/pbge/lunggens/CellRef/LungMapCellRef.html), scViewer-Lite, and ShinyCell for CellRef human (https://app.lungmap.net/app/shinycell-human-lung-cellref) and mouse lung (https://app.lungmap.net/app/shinycell-mouse-lung-cellref). These tools provide highly interactive search, analyzing, and visualization functionalities for users to explore and reanalyze cell type and gene expression patterns provided by the LungMAP CellRefs (Fig. 4, Supplementary Fig. 6). The LGEA CellRef page enables users to perform “Gene Expression Query”, “Cell Type Query”, and “Cell Signature Query”. The “Gene Expression Query” enables users to input any gene of interest to visualize the expression patterns and associated statistics in UMAP, Box, Notched Box, Beeswarm, Scatter plot, and bi-directional bar charts (Fig. 4A, B). The “Cell Type Query” enables users to select any one of the pre-defined cell types and obtain cell-type information collected by LGEA including cell selective marker genes, transcription factors, and surface markers, ligands and receptors) as well as a link to the LungMAP CellCards^29^ (Fig. 4C). The “Cell Signature Query” function provides cell type selective signature genes identified using the LungMAP CellRefs, along with interactive tables and bar graph that enables users to search differential expression statistics and compare the mean gene expression across all cell types (Fig. 4D). scViewer-lite is a R shiny based app that allows for comparative viewing of gene expression and/or other meta data overlapped on dimension reduction plots and violin plots. Users can also select and highlight cells of interest (Supplementary Fig. 6). In addition to these two newly developed web interfaces, LungMAP CellRefs can be interactively explored in LungMAP web portal using ShinyCell^32^ based web interfaces (https://lungmap.net/cell-cards, “CellRef scRNA-seq” tab). CellRef-ShinyCell allows cell-cell or gene-gene comparison and gene co-expression analysis, more importantly, we incorporated age and sex variables into the CellRef-ShinyCell App, to enable users to depict the age/sex-dependent gene expression in UMAP and cell-type distributions.

### Automated cell type annotation using the LungMAP CellRefs

**Fig. 5:**
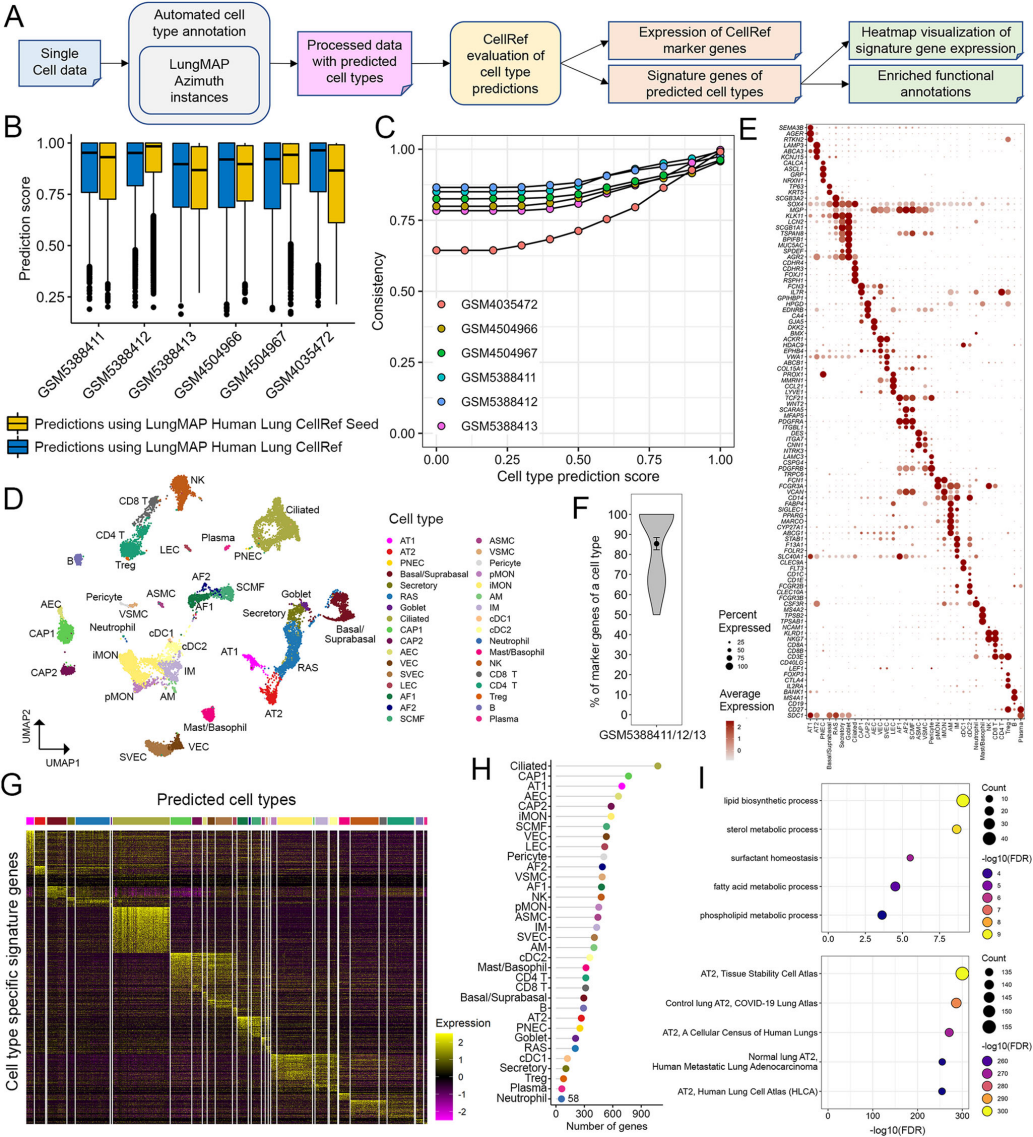
Cell type annotation and evaluation using the LungMAP Human Lung CellRef. **A** Schematic workflow of the automated cell type annotation and evaluation pipeline. **B** Distributions of cell type prediction scores in each test data. Prediction scores using CellRef Seed (yellow bars) are comparable to those using the complete CellRef (blue bars). Prediction scores (between 0 and 1) were calculated by the Seurat v4 MapQuery function for each cell. Box center lines, bounds of the box, and whiskers indicate medians, first and third quartiles, and minimum and maximum values within 1.5×IQR (interquartile range) of the box limits, respectively. GSM5388411: 6228 cells, GSM5388412: 8329 cells, GSM5388413: 7143 cells, GSM4504966: 8381 cells, GSM4504967: 8043 cells, GSM4035472: 5767 cells. **C** Consistency of cell type predictions using the CellRef Seed and CellRef in each test data. Consistency percentages (y axis) were calculated for cells in each test dataset (color) passing different thresholds of prediction scores (x axis). **D**–**H** Evaluation of automated cell type annotations for three of our test data (GSM5388411/12/13, three scRNA-seq of normal human lungs). Evaluation of the other three test data samples were shown in Supplementary Fig. 7. Basal and suprabasal cells were combined in prediction. **D** UMAP visualization of cells with prediction scores ≥ default cut-off (mean-1 standard deviation) and predicted annotations with at least 5 cells. Cells were colored by automated cell type annotations using the CellRef Seed as reference. Data from different donors were integrated using Seurat’s reciprocal principal components analysis (RPCA) pipeline. **E** Evaluation of cell type annotations using CellRef cell type markers from Supplementary Data 2. **F** Percentages of cell type markers (Supplementary Data 2) that are differentially expressed in their corresponding cell type predictions (*n* = 34 cell types) in (**D**). Data are shown using violin plot with dot and error bars representing mean ± SEM. **G** Heatmap visualization of expression of cell type specific differentially expressed genes (DEGs). **H** The number of DEGs for each predicted cell type. **I** Significantly enriched functional annotations using DEGs of the predicted AT2 cells: most enriched Gene Ontology Biological Processes (top) and ToppCell Gene Sets (bottom). Functional enrichment analysis was performed using ToppGene (https://toppgene.cchmc.org/enrichment.jsp). The minimum false discovery rate (FDR) was set to 1e−300. Please see Fig. 2 for definitions of cell type abbreviations.

We developed LGEA, ShinyCell, and scViewer-lite-based web interfaces for users to explore and analyze expression patterns of normal lung cells and genes of interest without the need for computational coding. Another powerful use of the LungMAP CellRefs is to use them for automated cell type annotation of users’ own single-cell datasets to facilitate analysis and standardization of cell type prediction and annotations. To achieve this goal, we built our CellRef Seeds and CellRefs into R objects in accordance with Seurat reference mapping pipeline^5, 20^. Azimuth (https://satijalab.org/azimuth) instances were established at LungMAP.net (https://lungmap.net/cell-cards, “CellRef Azimuth” tab) to enable users to upload their own datasets for online automated cell type annotation using our CellRefs (Fig. 5A) and exploration of any gene features on the projected UMAP or in Violin plots. Additionally, to facilitate evaluation of automated cell type annotation results, we developed functions in our R pipeline to visualize the expression of CellRef markers across all predicted cell types, identify cell type signature genes and their associated functional annotations, and compile all visualization and evaluation results into a single evaluation report using R markdown (Fig. 5A).

### Use cases driven evaluation of LungMAP single-cell references for automated cell type annotation

Annotation of scRNA-seq of normal human lung. We collected published scRNA-seq datasets of normal human lung samples to independently evaluate the accuracy of the automated cell type annotation using the LungMAP CellRefs. The datasets were from normal human lung samples of 2 months to 45 years of age and were generated using 10X chromium 3’ (GSM5388411/12/13^33^ and GSM4504966/67^34^; aligned to hg38 reference genome) and 5’ (GSM4035472^1^; aligned to hg19 reference genome) platforms.

For each test data, we used both the human lung CellRef Seed and CellRef to predict cell type annotations using Azimuth’s reference mapping algorithm^5,20^. More specifically, we used the Seurat v4 FindTransferAnchors and MapQuery functions. During the reference mapping, a prediction score (between 0 and 1) was calculated for each cell, reflecting the confidence associated with the predicted cell annotation. By default, we used the mean value minus one standard deviation as the cutoff for the prediction score; cells within the threshold are considered to be confidently mapped to the CellRef annotations. Using this cutoff, 80.28% cells in the test dataset can be confidently annotated using the CellRef in comparison with 80.84% using the CellRef Seed, suggesting that similar numbers of cells can be confidently predicted using both the complete human lung CellRef and the CellRef Seed (Fig. 5B, C). Predictions using the CellRef Seed were computationally efficient, taking ~1 min to annotate a 10x chromium scRNA-seq of 4000–8000 cells.

We evaluated CellRef performance via multiple independent approaches including using inter-dataset (external data) and intra-dataset (10-fold cross validation within CellRef samples) or based on prior knowledge (i.e., known markers and gene ontology terms). First, we applied the validation functions in our pipeline (Fig. 5A, “Methods”) to evaluate the accuracy of cell type predictions based on prior knowledge. As shown in Fig. 5D–I and Supplementary Fig. 7, predicted cell types were well separated and formed clusters. Cell-type-specific marker genes from CellCards were selectively expressed in each predicted cell type, supporting the concordance of the cell identities (Fig. 5E, F, Supplementary Fig. 7). Cell type specific signature genes were identified using widely accepted criteria (adjusted *p* value of Wilcoxon rank-sum test <0.1, expression frequency >=20%, and fold change >=1.5) (Fig. 5G, H). Functional enrichment analysis of cell type signature genes was used to further validate the predicted cell identities. For example, predicted AT2 cells were functionally enriched in “surfactant homeostasis” and “lipid/phospholipid/fatty acid metabolic processes” (Fig. 5I, top). ToppCell (https://toppcell.cchmc.org/) analysis showed that the predicted signature genes were consistent with genes selectively expressed in normal AT2 cells identified in independent single-cell studies of human lung^35,36^ (Fig. 5I, bottom). Next, we evaluated the CellRef performance using external datasets GSM5388411/12/13^33^. After mapping and comparison of the CellRef prediction to the original published cell type annotations, we measured the CellRef performance based on multiple metrics, including precision, recall, accuracy, F1 score, and Matthews correlation coefficient (MCC) (“Methods”). The median values of all metrics are greater than 0.92 (Supplementary Fig. 8), supporting the high consistency of the automated CellRef cell type prediction with the original cell annotations. Last, we performed a 10-fold cross validation of cell type identification within both human and mouse LungMAP CellRefs (“Methods”). Briefly, we randomly partitioned the data in the CellRef into 10 similar parts, used 9 parts as training data to predict cell types in the remaining part and measured the performance of the predictions based on F1 score and Matthews correlation coefficient (MCC) which quantified the consistencies of the predicted identities with the CellRef identities of the test part. We repeated the training and testing 10 times and used a different part as the testing data each time. Both the LungMAP human and mouse lung CellRefs achieved high cross validation performance with ~0.92 median F1 and MCC scores for the human lung CellRef and 0.98 median F1 and MCC scores for the mouse lung CellRef, respectively (Supplementary Fig. 9).

In summary, evaluations based on prior knowledge or using inter-dataset and intra-dataset demonstrate the high performance and accuracy of human and mouse lung CellRef cell type annotations, support the general applicability of automated CellRef cell type annotation for new data from scRNA-seq of lung.

Among the testing datasets, GSM4035472 showed relatively lower consistency score than others (Fig. 5C). This is a special dataset in two ways. It was generated using 10X Single Cell 5’ assay while other testing samples were using 10X Single Cell 3’ assays. This dataset was aligned using hg19 while others used hg38. We included this dataset for a proof-of-concept that CellRef can map cell types for datasets from different protocol and reference versions. The difference is likely due to the combination of different library and reference genome versions. Nevertheless, more than 75% cells from this dataset can be consistently mapped using the CellRef and CellRef Seed when using the default cutoff (Fig. 5C).

**Fig. 6:**
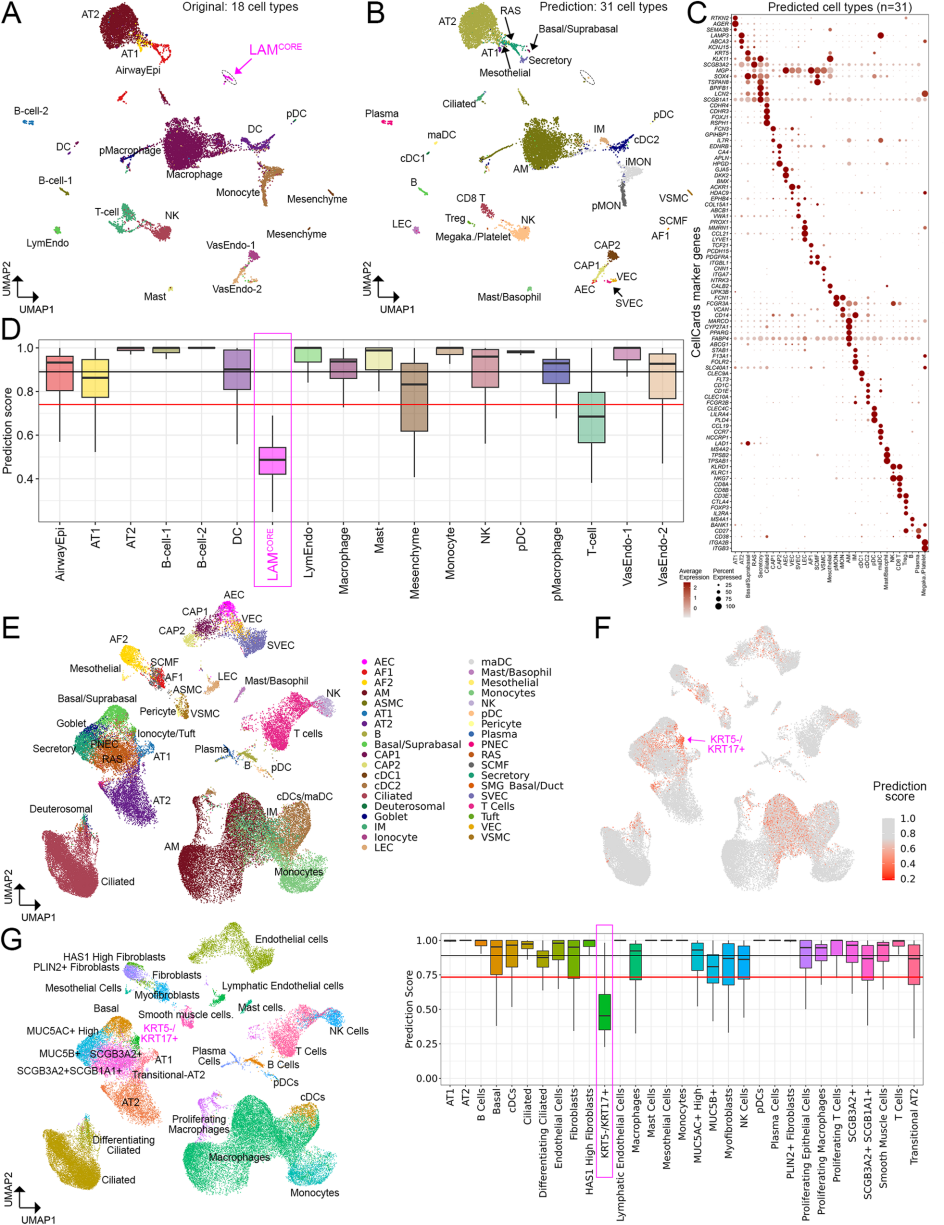
Application of LungMAP Human Lung CellRef to disease lungs. **A** UMAP visualization of a published scRNA-seq of human lungs with LAM^1^. Cell colors represent cell identities predicted in Guo et al., 2020, including a unique disease-related cell population, named LAM^CORE^ cells (magenta cell cluster). **B** UMAP visualizations of cells predicted using the CellRef Seed as reference. Basal and suprabasal cells were combined in the prediction. Prediction scores (between 0 and 1) were calculated by the Seurat v4 MapQuery function for each cell. Cells with prediction score >= the default cutoff (i.e., the mean minus 1 standard deviation value) were shown. Three singleton cell type predictions were not included. **C** Evaluation of cell type predictions using expression of representative CellRef marker genes. Megaka./Platelet: Megakaryocyte/Platelet. **D** Distributions of the cell type prediction scores in each of the original cell identities (*n* = 18 cell types; abbreviations were defined in Guo et al.^1^). The black and red horizontal line represents the mean and (1 standard deviation lower than the mean) value of the prediction scores, respectively. **E**–**G** UMAP and boxplot visualizations of application of CellRef to a published scRNA-seq of human lungs with idiopathic pulmonary fibrosis (IPF)^11^. **E** UMAP visualization of cells predicted using the CellRef Seed. Basal and suprabasal were combined, T cell subsets, and monocyte subsets were combined in the prediction. **F** UMAP visualization of cells colored by the prediction scores. **G** Left: UMAP visualization of cells colored by the original cell identities (*n* = 31 cell types; abbreviations were defined in Habermann et al.^11^). Right: boxplot visualization of the distribution of prediction scores in each of the original cell identities. The black and red horizontal line represents the mean and (1 standard deviation lower than the mean) value of the prediction scores, respectively. The disease-associated KRT5-/KRT17+ cells had prediction scores below the cutoff line. The number of data points in each boxplot in (**B**) and (**G**) can be found in Fig. 6 source data table. In (**D**) and (**G**), Box center lines, bounds of the box, and whiskers indicate medians, first and third quartiles, and minimum and maximum values within 1.5×IQR (interquartile range) of the box limits, respectively. Please see Fig. 2 for definitions of CellRef cell type abbreviations.

Application to scRNA-seq of human lung diseases. We previously performed single-cell transcriptomic analyses of lung samples from patients with lymphangioleiomyomatosis^1^ and identified a unique population of cells termed LAM^CORE^ that were readily distinguished from endogenous lung cell types and shared closest transcriptomic similarity to uterine myocytes in both normal and LAM uteri^1^. In the present work, we re-aligned this dataset to the hg38 reference genome and performed automated cell type annotation using the LungMAP Human Lung CellRef Seed. A total of 31 cell types were predicted from the two LAM lungs (Fig. 6A–C) in comparison with 18 cell types predicted from the original publication^1^. Cell type predictions were largely consistent with the original clustering-based annotations^1^, but more cell subtypes not reported in the original study can be distinguished using CellRef. Importantly, the previously identified LAM^CORE^ cells (73 cells) had the lowest average prediction score below the cutoff line (Fig. 6D), supporting the notion that this LAM^CORE^ cell population was not similar to normal lung cell types in the present LungMAP CellRef.

In the second use case, we used an idiopathic pulmonary fibrosis (IPF) lung scRNA-seq dataset (GSE135893, 10X Single Cell 5’, 19 samples, 12 IPF lungs) (Fig. 6E–G). Habermann et al. reported the identification of 31 cell types including a previously unrecognized KRT5-/KRT17+ pathologic, ECM-producing epithelial cell population that was highly enriched in IPF lungs^11^ (Fig. 6G, left panel). Using this data, CellRef predicted 37 cell types and identified more endothelial and rare epithelial cell subtypes (e.g., PNEC and ionocytes) (Fig. 6E). Most importantly, the median prediction score of KRT5-/KRT17+ cells was the lowest among all cells (Fig. 6F, G, right panel); significantly lower than our default cutoff threshold, suggesting that this cell population cannot be confidently mapped to any of the normal lung cell types in CellRef and likely represents an atypical or pathogenic cell population. In summary, these use cases provide proof-of-principle examples that CellRef can be used to assist analysis of lung disease data and identify potential disease-related cell clusters. Further morphological analyses and functional validations are then needed to identify and characterize any abnormal cell types or atypical cell states.

### Benchmark analysis of cell type accuracy and stability of the LungMAP Human Lung CellRef

In addition to CellRef, prior healthy lung cell atlases have been reported, including Travaglini et al.^15^ which included data from 3 human lungs, and the recently released integrated version of Human Lung Cell Atlas (HLCA)^37,38^. The HLCA core reference was used, which defined 58 lung cell types/states based on an integration of single-cell RNA-seq data from 167 healthy samples from 107 individuals from 14 datasets^37^.

**Fig. 7:**
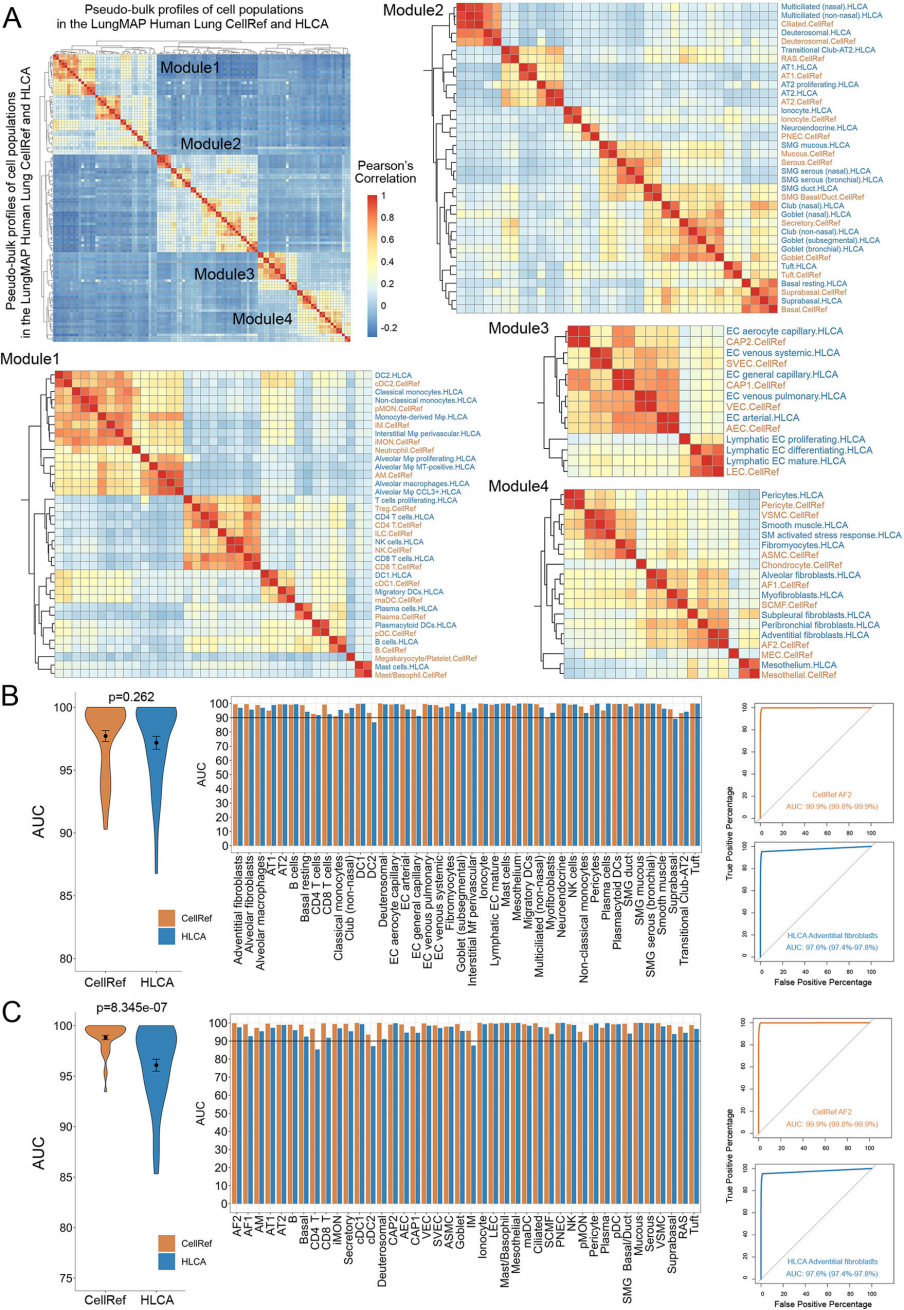
Assessment of cell type prediction accuracy of the LungMAP Human Lung CellRef. **A** Heatmap visualization of Pearson’s correlations of cell types between the human lung CellRef and the Human Lung Cell Atlas (HLCA)^37^. A pseudo-bulk profile was created for each cell type of either CellRef or HLCA by averaging each gene’s expression in the cells of the cell type. Cell types were clustered into four modules, each corresponding to one of the four major cell-lineages. Correspondences of CellRef and HLCA cell types within each of the four modules were shown based on the hierarchical clustering analysis. **B**, **C** Assessment of cell type accuracy based on marker gene expression. **B** Area under the receiver operating characteristic (ROC) curve (AUC) values for each of the mapped cell types (*n* = 42) in CellRef (orange) and HLCA (blue) calculated using the cell type selective marker genes identified from the HLCA study. Left: summary of the AUC values using violin plots. Middle: AUC values for each of the mapped cell types. Right: using CellRef AF2 (HLCA adventitial fibroblasts) as an example to show the ROC curves labeled with AUC values and 90% confidence interval. **C** AUCs values for each of the mapped cell types (*n* = 42) in the CellRef (orange) and HLCA (blue) calculated using the cell type selective marker genes identified by CellRef (Supplementary Data 5). Left: summary of the AUC values using violin plots. Middle: AUC values for each of the mapped cell types. Right: using CellRef AF2 (HLCA adventitial fibroblasts) as an example to show the ROC curves labeled with AUC values and 90% confidence interval. In both (**B**) and (**C**), the black dot and error bars represent mean ± SEM. *p* value represents significance of difference assessed using two-tailed paired Welch’s *t* test. CellRef cell type abbreviations are described in Fig. 2.

To assess and benchmark the accuracy of cell type identification and marker genes prediction, we compared LungMAP human lung CellRef with HLCA using multiple independent approaches. First, we accessed the overall similarity/distinction of the two atlases based on correlation analysis of pseudo-bulk expression of highly variable genes (see “Methods” for details). Although the cell type names are not identical, the correlations of cell type pseudo-bulk profiles between HLCA and CellRef were highly consistent among the four major lung cell lineages (Fig. 7A). Within each lineage, most CellRef and HLCA cells have a one-to-one mapping (Fig. 7A). In addition, each reference identified several unique lung cell types or states (i.e., cell types do not cluster together between the two references). Among these, the chondrocytes, myoepithelial (MEC), ILC, Treg, and neutrophil cells were unique in our CellRef, while subpleural fibroblast, AT2 proliferating, and T cell proliferating were unique in HLCA. We summarized the one-to-one mapped cell types and the unique cell types/states in the Supplementary Fig. 10. In summary, the consensus across the two lung atlases is very high, providing further assurance of accuracy. By identification of common and unique cell identities across the two atlases, our efforts represent an initial standardization step to begin mapping a complex cell group with multiple names across different lung atlases.

Next, we assessed and compared the accuracy of cell type identification and cell selective marker prediction via receiver operating characteristic (ROC) curve analysis of cell type selective marker genes ranking expression in the predicted cells, a method we developed and incorporated into SINCERA pipeline^30^ (“Methods”). Using this approach, we performed the single-cell ranking based on the expression of the marker genes of each cell type and compared the rankings against the cell type annotations in reference to obtain an area under ROC curve (AUC) for each cell type. A higher AUC represents a higher accuracy of cell type identification in a reference. To ensure the fair comparison, we first calculated the AUCs for mapped cell type pairs between the two references using the cell type marker genes identified by HLCA. As shown in Fig. 7B, the average AUC of 97.7% is achieved for CellRef, slightly higher than 97.2% for HLCA but with no significant difference (*P* value = 0.262). Next, we performed the same analysis using the cell type marker genes identified by CellRef (Supplementary Data 5). The results were consistent (CellRef: 98.8%; HLCA: 96.1%) (Fig. 7C). Using the CellRef markers, CellRef out-performed HLCA in this case (*P* value = 8.345E−07) (Fig. 7C).

**Fig. 8:**
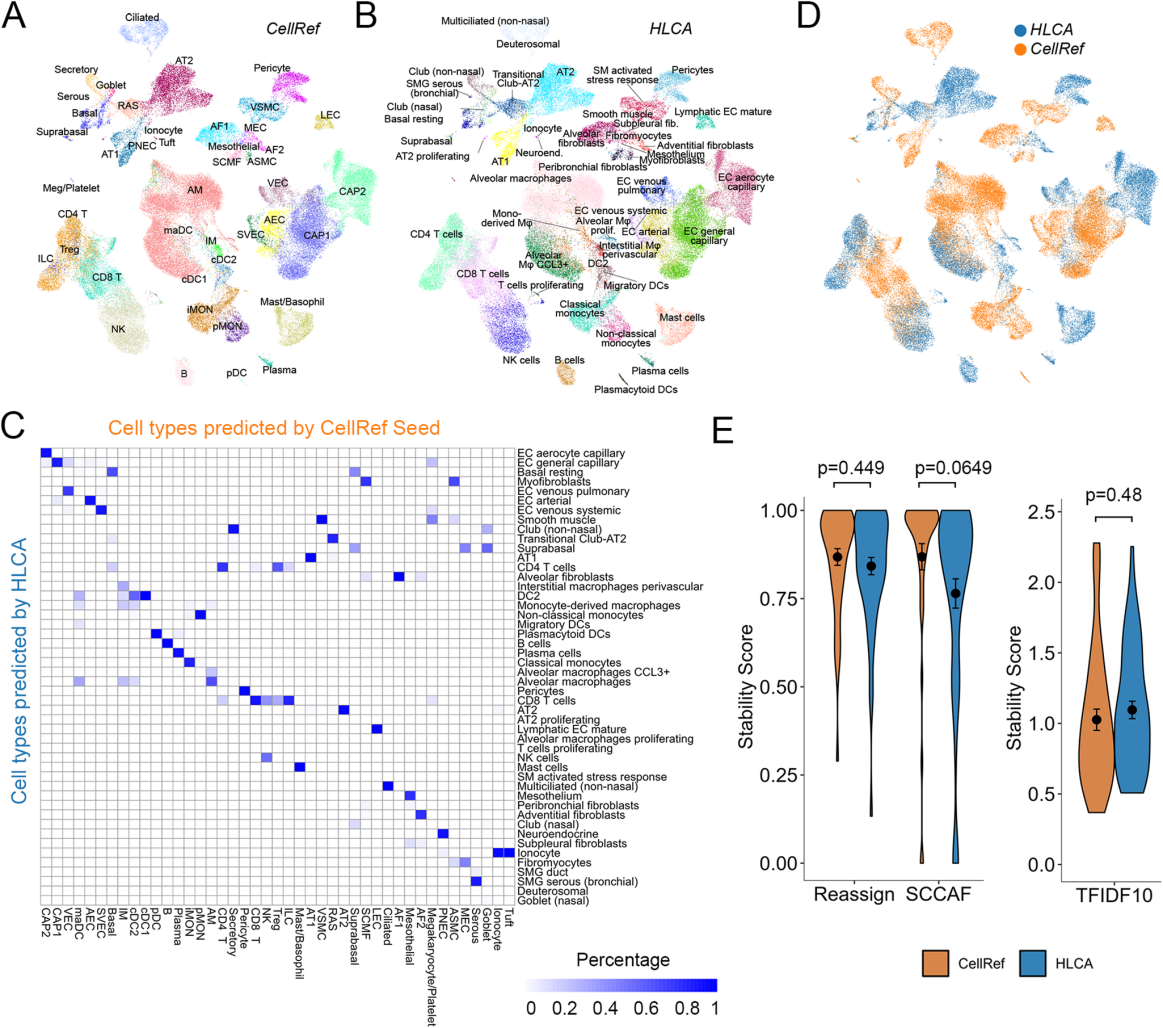
Assessment of cell type stability of automated annotation using CellRef. **A**, **B** UMAP projection of scRNA-seq (Travaglini et al.^15^, *n* = 3 human lungs) with Azimuth projected cell type annotations using the LungMAP Human Lung CellRef Seed (**A**) or using the Human Lung Cell Atlas (HLCA)^37^ (**B**) as the reference. **C** Corresponding cell-population assignments of CellRef and HLCA (mapping percentage relative to CellRef). **D** Cells colored by “winning” annotations from CellRef or HLCA determined by scTriangulate based on stability assessments (shown in **E**) annotations. **E** Violin plot visualization of stability metric scores calculated using scTriangulate, including reclassification accuracy (SCCAF and reassign) or marker gene specificity (TF-IDF score), for all Azimuth assigned CellRef or HLCA cell populations (*n* = 42 cell populations predicted using the CellRef Seed; *n* = 48 cell populations predicted using HLCA) in Travaglini et al. 2020. The black dots and error bars represent mean ± SEM. *p* value represents significance of difference assessed using two-tailed unpaired Welch’s *t* test. Please see Fig. 2 for definitions of CellRef cell type abbreviations.

Next, we assessed and compared performance of CellRef and HLCA using the data from first version of human lung atlas^15^ as the test data. After the mapping using the CellRef Seed and HLCA, we found that 43 out of 48 (89.6%) CellRef and 48 out of 58 (82.8%) HLCA cell types were predicted and most of the predictions had a one-to-one mapping (38 out of 43 CellRef clusters) (Fig. 8A–D). Disagreements include CellRef interstitial macrophages (IM) which was subdivided into HLCA monocyte and DC subsets and CellRef suprabasal cells was subdivided into HLCA Basal resting and suprabasal cells. Vice versa, HLCA myofibroblasts was subdivided into SCMF and ASMC in CellRef prediction; HLCA CD8 T cells were subdivided into CD8 T, NK, Tregs, and ILC in CellRef prediction; platelets and Tuft cells were only predicted by CellRef while some alveolar macrophage subtypes such as monocyte-derived macrophages, alveolar macrophage proliferating were only predicted by HLCA. Hence, all three versions of Lung Atlas are highly consistent, with the different atlases providing potentially diverse resolution levels resulting in some discrete lung cell populations.

To quantitatively assess the validity of the cell population predictions between CellRef and HLCA, we applied three distinct cluster stability measurements in the recently published single-cell integration framework scTriangulate^18^. In brief, scTriangulate aims to compare the biological stability of conflicting clusters amongst multiple annotations, such that each single cell can be assigned to the most stable annotation. The stability metrics include reclassification statistics (SCCAF)^39^, centroid-based reassignment (Reassign)^18^, and marker gene specificity (Term frequency - Inverse Document frequency or TF-IDF)^18^. SCCAF and Reassign metrics measure whether the atlas leads to definitive cell population predictions and with high confidence (i.e., cells can be reproducibly re-classified to these clusters). A higher TFIDF score suggests there are more unique/informative markers associated with the defined cluster. Applying these stability metrics to CellRef and HLCA on the test data from Travaglini et al., we observed that, on average, CellRef produced slightly higher SCCAF and Reassign scores as compared to HLCA; while HLCA produced slightly higher TF-IDF score than CellRef (Fig. 8E). Similarly, when applying these stability metrics to CellRef and HLCA using their own data collections, CellRef produced slightly higher scores on SCCAF and TF-IDF; as well as a significantly higher score on cell re-assignment (*p* = 0.011) as compared to HLCA (Supplementary Fig. 11). scTriangulate leverages the computed stability scores to further assess the marginal importance of each annotation (CellRef and HLCA) using a cooperative game theory framework. scTriangulate predicted that 50% (*n* = 24/48) of the CellRef cell types to be of higher confidence (AEC, AF1, AF2, AM, ASMC, CD8 T, cDC1, cDC2, Ciliated, Goblet, Ionocyte, LEC, Platelet, pMON, Suprabasal, SVEC, VSMC), compared to 46% (*n* = 27/58) of the HCLA cell types (AT2, aCAP, Pericyte, Mesothelium, Peribronchial fibroblasts and Adventitial fibroblasts, etc.) (Fig. 8D). Hence, both atlases provide unique and informative predictions, each with distinct benefits.

Next, we compared data integration in the human lung CellRef and HLCA using Local Inverse Simpson’s Index (LISI)^17^, which calculates two metrics for each cell in the integration: (i) iLISI that measures the number of data batches in the neighborhood of each cell, and (ii) cLISI that measures the number of cell types in the neighborhood of each cell. Based on the assumption of LISI, iLISI scores are close to the batch numbers for a well mixing of balanced data batches, meanwhile the ideal cLISI score is close to 1 for a well separation of cell types in the integration. Since the total number of batches in CellRef and HLCA are different, for each reference, we calculated the iLISI scores for cells within each cell type and normalized the scores by the total number of batches in each cell type. The overall distributions of iLISI scores in both CellRef and HLCA are not ideal, on average about 15% for CellRef, and 5% for HLCA, respectively (Supplementary Fig. 11), likely due to that both atlases were constructed using large collections of heterogeneous data from different biological regions and conditions rather than a well-designed balanced cohort. We speculate whether LISI is a suitable approach to evaluate large-scale heterogenous single-cell data integration. Nevertheless, CellRef consistently outperforms HLCA on the measurements of iLISI, and has the cLISI scores close to 1, indicating a well separation of the CellRef cell types (Supplementary Fig. 11).

Last, we evaluated the LungMAP mouse lung CellRef using the three stability metrics calculated by scTriangulate^18^ and the marker-based AUC analysis^30^ (“Methods” and Supplementary Data 6). Results supported the high accuracy, cluster stability, and marker specificity of our mouse lung reference cell types (Supplementary Fig. 12; mean AUC 95.8, mean SCCAF score: 0.970, mean Reassign score: 0.881, mean TFID10 score: 0.892).

## Discussion

In the present study, we developed a computational approach to integrate large scale and heterogeneous sc/snRNA-seq datasets and constructed comprehensive lung single-cell references, termed LungMAP Human Lung CellRef and LungMAP Mouse Lung Development CellRef, in accordance with a well-defined cell type dictionary derived from LungMAP CellCards^10^. Evaluation functions were developed in our pipeline to perform fast and comprehensive evaluation of the predicted cell type annotations. User-friendly web interfaces were developed to facilitate access, visualization, and utilization of the LungMAP CellRefs. For advanced users who are interested in annotating their own datasets using the LungMAP CellRefs, we established Azimuth instances to support online automated cell type annotations of users’ own scRNA-seq or independently produced compendiums. Regarding the choice of the classification algorithm (Azimuth), this algorithm leverages Seurat’s label transfer method, which performs well in prior diverse benchmarking evaluation studies^40^ and has been broadly used. Importantly, it is currently the fastest and most accessible approach for reference-based label transfer, data pre-processing and exploration, and is compatible with datasets containing hundreds of thousands of cells processing in just a few minutes. LungMAP plans to update the specific version of Azimuth as it is updated in the future.

The LungMAP Human Lung CellRef contains a total of 347,970 cells and 48 well-defined lung cell types, covering major cellular heterogeneity in the four regions: trachea, bronchi, SMG, and lung parenchyma. The CellRef identified cell types mapped to the cell type nomenclature in the LungMAP CellCards^10^. In addition, based on unbiased clustering analysis, we identified cell types that are not yet included in the CellCards but reported in recent scRNA-seq analyses, including deuterosomal cells^14^, suprabasal cells^14^, systemic venous endothelial cells^21^, mature dendritic cell subset, SMG duct cells, respiratory airway secretory cells (RAS, a recently identified multipotent secretory cell population in respiratory bronchioles), and megakaryocyte/platelets^15,22^. During the CellRef construction, we discovered cell clusters selectively expressing marker genes of these new cell types, and thus we have included these cell types into the LungMAP Human Lung CellRef. We will continue to incorporate more cell types in accordance with new findings from single cell and/or functional studies.

To our best knowledge, two earlier versions of human lung references^15,37^ have been published or are in preprint. We compared and incorporated the first lung reference into our CellRef construction. Further, we carefully compared all annotated cell types in the recently released integrated version of Human Lung Cell Atlas (HLCA) with LungMAP CellRef based on the highly variable genes from HLCA and CellRef. Although not all cell type names are identical, the majority of the HLCA annotated cells align well with a clearly defined cell type in LungMAP CellRef. Furthermore, each reference identified several unique lung cell types or states (i.e., cells that don’t align to any given cell cluster in the other reference). In addition, we performed a series of benchmark studies to compare the two integrative lung atlases including ROC-based analyses to cross-validating the accuracies of shared cell type identifies in both references and used scTriangulate^18^, a recently described cluster stability assessment framework to quantitatively assess and compare the cluster stability of the two atlases based upon three independent statistical metrics. We found that cell type predictions using CellRef and HLCA were highly consistent, with discrete and stable populations in both atlases. Each reference had approximately an equal percentage of cell type predictions that were more confident in one than the other. Hence, both atlases provide unique and informative predictions, with benefits to each atlas. Generating a consensus blueprint of normal human lung with unified cell ontology and nomenclature is fundamentally important and challenging, requiring cross consortia efforts and open discussions among the pulmonary research community at large. Our efforts herein represent the beginning of initiatives to build a consensus atlas by mapping a complex cell group with multiple names across different lung atlases. Further cross-team discussions and comparisons are needed to reach the ultimate goal of a unified nomenclature and standardized data processing that are needed to create an enduring resource for the research community.

The present LungMAP CellRefs has several unique features: (1) We developed a computational pipeline and a guided approach to construct and evaluate the reference which can be reused for future updates of LungMAP CellRef or references of other organs; (2) The LungMAP CellRef identifies cell types in accordance with the LungMAP CellCards^10^, a rigorous catalog of lung cells validated by both single cell and functional studies. The CellCards^10^ curation effort, is now a pan-consortium effort which includes multiple laboratories outside of LungMAP to define standardized cell populations, labels, markers, and functional descriptions, leveraged by CellRef. Thus, we consider CellRef a more knowledge driven as opposed to solely cluster driven, which in our view represents a sustainable and reliable model; (3) During CellRef construction, we identified the best seed populations for each cell type (CellRef Seeds), which was not only used to construct the complete CellRef but can be independently used for automated cell type annotation and online visualization with improved computational efficiency and hardware requirements; (4) We constructed LungMAP CellRef for both human and mouse, the two most commonly used species, and provide options for users to use either scRNA-seq or snRNA-seq based CellRef separately based upon the input sequence type to achieve better performance on cell type annotation; (5) Web portals were developed by our LungMAP research centers and data coordination center to facilitate resource sharing and maximize use of the constructed references by the research community.

While these efforts illustrate the power of reference-guided classification from a comprehensive reference, we note there are still several areas for improvement in both CellRef and independent initiatives. First, the current iteration of CellRefs does not yet clearly define the spectrum of possible immune sub-populations and transitional cell states. Such populations are likely to vary with age and disease. Antibody-based approaches, e.g., CITE-seq or flow cytometry, are likely to aid in the annotation of lung immune cell sub-populations^41^. Future lineage/compartment specific reference constructions will be useful in providing enhanced resolutions and granularity at sub-cell type and cell transitional states levels. Further, the current data collections do not have sufficient statistical power for precise annotation of certain rare lung cell types, e.g., SMG duct cells. Region specific Laser Capture Microdissection (LCM) and cell sorting will be useful in identifying and capturing rare lung cell types and their RNA expression patterns. Finally, there are unique disease-associated cell-populations, including infiltrating cell populations, which will likely necessitate the classification and inclusion of disease specific cell populations and cell-states^1,11^. Thus, data integration and annotations across independently derived normal healthy and disease atlases or clustering solutions are a likely a new direction for future atlas efforts.

In summary, we developed a computational pipeline utilizing a cell type dictionary to consolidate single-cell transcriptomic datasets and constructed LungMAP CellRefs and CellRef Seeds for normal human and mouse lung. CellRef Seed has an equivalent prediction power and produces consistent cell annotation as the full CellRef, but with significantly improved computational efficiency and hardware requirements; facilitating utilization for automated cell type annotation and online visualization, addressing a significant computational challenge for single-cell reference applications. Using independent datasets, we demonstrated the utility of CellRefs for automated cell type annotations of normal lung and for potential identification of disease-related cells based on their deviation from normal pulmonary cells. Our CellRefs, along with the developed analytic and web-based tools, are freely available to the pulmonary research community to facilitate hypothesis generation, research discovery, and identification of cell type alterations in disease conditions.

## Methods

### Ethical approval

UPenn LungMAP cohort used samples from de-identified non-used lungs donated for organ transplantation via an established protocol (PROPEL, approved by University of Pennsylvania Institutional Review Board) with informed consent in accordance with institutional and NIH procedures, and provided by next of kin or healthcare proxy. All patient information was removed before use. This use does not meet the current NIH definition of human subject research, but all relevant guidelines and regulations and all institutional procedures required for human subject research were followed throughout the reported experiments. CCHMC LungMAP cohort used de-identified human bronchus samples provided by the Marsico Lung Institute Tissue Procurement and Cell Culture Core at the University of North Carolina, Chapel Hill, NC (UNC) from lung transplant organ donors. Participants did not receive monetary compensation and consent was obtained by United Network for Organ Sharing affiliated Organ Procurement Organizations (UNC Office of Human Research Ethics protocol # 03-1396). For mouse study, animal protocols (2C12114, 2015-0060, 2018-0072, and 2021-0053) were approved by the Cincinnati Children’s Hospital Medical Center Institutional Animal Care and Use Committee in accordance with NIH guidelines.

### Collection and pre-processing of single cell/single nucleus RNA-seq of human lung

We collected eight published and two unpublished sc/snRNA-seq datasets of human lung for LungMAP human lung single-cell reference construction. For the published datasets, unique molecular identifier (UMI) count matrix of gene expression in single cells were downloaded from Gene Expression Omnibus (GEO), European Genome-phenome Archive (EGA), or Synapse.org using the following accession numbers: GSE122960^12^, GSE135893^11^, GSE134174^16^, GSE136832^2^, GSE161382^13^, EGAS00001004082^14^, GSE171524^3^, syn21041850^15^. For all datasets, hg38-alignment-based data from normal/control lung samples were used.

The CCHMC LungMAP cohort performed scRNA-seq experiments of human lung submucosal glands (SMG) obtained from five de-identified normal lungs. We isolated SMG tissue (~1 mm in long) from the human lung bronchus by microdissection under a stereo microscope (Leica M165 FC) using fine scissors and forceps, followed by dissociating the SMG in cocktail of prewarmed digestion solution of 0.2 mg/mL collagenase II (Thermo Fisher; cat. no. 1710105) and 0.1 mg/mL DNase I (Sigma-Aldrich; cat. no. DN25) in PneumaCult-EX medium (Stem cell technologies; cat. no. 05008) containing 1% Penicillin-Streptomycin (Thermo Fisher; cat. no. 15-140-163) for 30 min. The dissociated single cells were filtered using a strainer (100 µm; Corning; cat. no. 431752) and centrifuged at 300 × *g* for 5 min, the supernatant was discarded. The single cells were resuspended with Hanks’ Balanced Salt Solution (Thermo Fisher; cat. no. 88284) and analyzed using a 10x Single Cell 3’ v3 sequencing kit following the protocol provided by the company. Sequencing read alignment to the hg38 human genome and UMI-based gene expression matrix generation were performed for each sample using 10x Cell Ranger v5.

The normal samples used for the UPenn cohort in this study were from de-identified non-used lungs. scRNA-seq experiments (10x Single Cell 3’ v2 and v3 chemistry) were performed as described in Basil et al.^4^. In brief, pleura and visible airways/blood vessels were dissected away, mechanically minced into ~2 mm pieces, and processed into a single-cell suspension. After a single-cell suspension was obtained, cells were loaded onto a GemCode instrument (10x Genomics, Pleasanton, CA, USA) to generate single-cell barcoded droplets (GEMs) according to the manufacture’s protocol. The resulting libraries were sequenced on an Illumina HiSeq2500 or NovaSeq instrument.

#### Data pre-processing

For published datasets with original cell type annotations, we included cells selected in the original analyses. For published datasets without original cell type annotations (Reyfman et al.^12^) and unpublished datasets (UPenn LungMAP cohort and CCHMC LungMAP cohort), the following quality control (QC) criteria were applied to cell prefiltering, including 500–7500 expressed genes, less than 25% of UMIs mapped to mitochondrial genes, and less than 50,000 total UMIs. For scRNA-seq data from Donor29 in the CCHMC LungMAP cohort, we used 1500–7500 as the criterion for the “number of expressed genes” based on its unique cell distributions. After pre-filtering, Scrublet^42^ (v0.2.3) was performed to identify and remove potential doublet cells from each data sample. In total, 505,256 cells from 148 sc/snRNA-seq samples from 104 donors were used as input for our guided pipeline to construct the single-cell reference of normal human lung.

### Mice and Drop-seq of mouse lung development

C57BL/6J mice (Jackson Laboratories), embryonic days (E) 16.5, 18.5 to postnatal days (PND) 1, 3, 7, 10, 14, 28, were used for single-cell RNA-seq experiments using Drop-seq^43^. All mice were time mated. The presence of a vaginal plug was defined as E0.5. PND1 was defined as 24 ± 6 h after birth.

Lung dissection, single cell suspension, and Drop-seq library preparation of mouse lungs were described in the Methods of Guo et al.^22^. Data from PND1 was published in Guo et al.^22^. The alignment of paired-end sequence reads to mouse genome (mm10) and the generation of digital expression matrix were processed using Drop-seq tools (https://github.com/broadinstitute/Drop-seq/, v2.3.0) with default parameters. The expression matrix was generated by counting the number of unique molecular identifiers (UMIs) per gene per cell. In total, gene expression in 17 Drop-seq samples from eight time points (Supplementary Data 3) of mouse lung development were generated. For each data sample, the following pre-processing steps were performed. EmptyDrops^44^ in the Bioconductor package DropletUtils (v1.4.3) was used to identify cell barcodes with expression profiles significantly deviated from the profiles of empty droplets in each data sample with the parameters: lower = 100, FDR < 0.01. Filters were then applied to keep cells with 400–7500 genes, less than 40,000 UMIs, and less than 10% UMIs mapped to mitochondrial genes. Potential doublet cells in each sample were predicted and removed using Scrublet^42^. Ambient background RNAs were cleaned from gene expression in each cell using SoupX (v1.6.2) using contamination fractions automatically estimated from data.

### Guided construction of single-cell reference

Our guided single-cell reference (CellRef) construction workflow consists of four major steps: data integration, candidate cell cluster identification, seed cell identification, and consensus prediction for CellRef. We compiled a cell type dictionary containing a list of cell types and associated marker genes, including positive (selectively expressed in the cell type) and negative (no expression in the cell type) markers. We required at least two positive markers for each defined cell type to be included in our CellRef construction.

(i) *Data integration*. Multiple algorithms have been integrated into our R workflow, including mutual nearest neighbor (MNN) matching^45^, reciprocal principal component analysis (RPCA) in Seurat^20^ (v4.1.0), and Harmony^17^ (v0.1.0). By default, we use the align_cds function in Monocle 3 (v1.0.0) to perform MNN matching based data integration and batch correction. This is based on the UMAP inspection on the batch removal effects and cluster stability metrics measurement after applying different integration methods. Before integration, we merge data from all datasets into a single gene expression matrix, use it to construct a Monocle 3 cell_data_set object, and use the preprocess_cds function in Monocle 3 to normalize data to address read depth differences, regress out cell cycle effects and mitochondrial percentage differences, and calculate principal components representing major variances in the data.

(ii) *Candidate cell cluster identification*. Using the integrated data, we identify candidate cell clusters for each cell type listed in the dictionary using a combination of unbiased clustering algorithm and marker-based single-cell ranking. We perform unsupervised clustering analysis to group cells into distinct cell clusters based on transcriptomic similarity. By default, we perform clustering using the Leiden algorithm^46^ implemented in the cluster_cells function in Monocle 3.

Followed by the clustering analysis, we perform a “single cell ranking” for each cell type $i$ listed in the dictionary. Let ${P}_{i}$ be the set of positive marker genes of cell type $i$. For each marker gene $x\in {P}_{i}$, we identify ${Z}_{{xi}}$, a set of cells with positive (>0) zscore-scale expression of $x$, and generate ${R}_{{xi}}$, a ranking of cells in ${Z}_{{xi}}$ in the descending order based on zscore-scaled expression of $x$. We then aggregate all rankings $\left\{{R}_{{xi}}{|x}\in {P}_{i}\right\}$ into a single global ranking of cells, denoted as ${R}_{i}$, for the cell type $i$, aiming to identify cells that are ranked highly by multiple cell type marker genes. The aggregation was performed using an order-statistics-based robust rank aggregation algorithm, which assigns a score to each cell in ${R}_{i}$ to represent significance of the cell that is ranked consistently better than expected under a null hypothesis derived from $\left\{{R}_{{xi}}{|x}\in {P}_{i}\right\}$. Cells passing selection criteria were used as candidates for cell type mapping.

Using the clustering and single cell ranking results, we determine candidate cell clusters for each cell type $i$ as follows. Let ${\varphi }_{i}$ be the set of cells passed selection criteria (by default, significance score <0.1) cells in ${R}_{i}$ and $\sum$ be the cell clusters that we obtained from the unbiased clustering analysis. We calculate the precision and recall values for each cluster ${\sigma }_{j}\in \sum$ as follows: ${precision}\left(i,\, j\right)=\left|{\varphi }_{i}\cap {\sigma }_{j}\right|/\left|{\sigma }_{j}\right|$, ${recall}\left(i,\, j\right)=\left|{\varphi }_{i}\cap {\sigma }_{j}\right|/\left|{\varphi }_{j}\right|$, where $\left|{\varphi }_{j}\right|$ and $\left|{\sigma }_{j}\right|$ denote the number of cells in ${\varphi }_{j}$ and ${\sigma }_{j}$, respectively, and $\left|{\varphi }_{i}\cap {\sigma }_{j}\right|$ denotes the number of cells in both ${\sigma }_{j}$ and ${\varphi }_{j}$. The candidate cell clusters for cell type $i$ is determined as ${A}_{i}=\{{\sigma }_{j}\in \sum {|precision}\left(i,\, j\right)\ge F,\, {recall}\left(i,\, j\right)\ge S,{F}\in \left[{{{{\mathrm{0,1}}}}}\right],S\in \left[{{{{\mathrm{0,1}}}}}\right]\}$. By default, we use F = 0.05 and S = 0.25. A QC inspection of the candidate cell clusters is recommended to ensure the accuracy for the CellRef construction.

In summary, in step 2, we use unsupervised clustering in conjugation with marker-based single-cell ranking to select most relevant cell groups candidates. The use of unbiased clustering before seed cell identification can also provide an opportunity to discover new cell types that have not yet been defined in the dictionary. For example, if the marker genes of a newly reported cell type are co-selectively-expressed in our cell clusters, this new cell type and marker genes are added to the cell type dictionary and then included in the downstream seed cell identification and CellRef construction.

(iii) *Seed cell identification*. In this step, we aim to identify cells that best represent the identity of each cell type using single-cell ranking based on marker genes in the dictionary. These cells will then serve as seeds to construct the CellRef. For a cell type $i$, we first identify cells with expression of any negative markers of $i$ or expressed less than two positive markers of $i$ and remove those cells from ${A}_{i}$ (the candidate cell clusters of cell type $i$ that we identified in step 2). Using the remaining cells in ${A}_{i}$, we perform single cell ranking using the positive markers of $i$ as described in step 2 and generate an aggregated ranking of cells. Top-ranked cells in the aggregated list will be selected as the seed cells for cell type $i$.

(iv) *Consensus prediction*. Once all seed cells are identified, we use them to predict cell type annotations of all cells in the collection using two independent automated cell type annotation algorithms, Seurat’s label transfer^5,20^ and SingleR^6^ (v1.6.1). For the Seurat’s label transfer based prediction, we integrate scRNA-seq data of the “seed” cells using SCTransform normalization based reciprocal principal component analysis (RPCA) integration, perform SCTransform normalization on gene expression in each of our collected datasets, and predict cell type annotations using the MapQuery function in Seurat v4. A predicted cell type and an associated prediction score were assigned to each query cell based on transcriptomic similarity between the query cell and the “seed” cells. Cells with low prediction scores (by default, lowest 10%) were excluded from the CellRef construction. For the SingleR-based prediction, we normalize gene expression in the seed cells and in a query dataset by total UMIs per cell and use the SingleR function with default parameters to predict cell type annotations for the query cells. We removed poor-quality or ambiguous predictions using the pruneScores function. Let $Y$ be the set of cells with consistent cell type predictions in both methods. We calculated a *k*-nearest-neighbor purity (kNN-purity) metric for each cell in $Y$, measuring the percentage of the cell’s *k* nearest neighbors (by default, *k* = 20) that have the same cell type prediction. The complete CellRef was comprised of the seed cells and the cells that have consistent cell type predictions in both methods and with kNN-purity > =0.6.

### Construction of the LungMAP Human Lung CellRef

We constructed a cell type dictionary for normal human lung (a list of cell types and their associated marker genes) based on the cell types and marker genes listed in the LungMAP CellCards^10^. In addition, we extended the dictionary to include seven human lung cell types reported in recent single-cell studies but not yet in CellCards, including systemic venous endothelial cell (SVEC), deuterosomal cell, submucosal gland (SMG) duct cell, megakaryocyte/platelets, suprabasal cell, mature dendritic cell (maDC), and respiratory airway secretory cell (RAS). In total, 48 cell types are defined in the dictionary.

Using this cell type dictionary, we performed the guided CellRef construction described above using seven scRNA-seq datasets. The original data were aligned to three versions of 10x Cell Ranger hg38 reference genome. To reduce the impact of reference genome differences on the data integration, we used the expression of 32,278 common gene features (based on Ensembl IDs) among the three reference genome versions to perform data integration (considering 104 donors as individual batches) and candidate cell cluster identification as described above. A curation was performed on the candidate cell cluster assignment by inspection of marker genes expression in the cell clusters. Based on the curated candidate cell clusters for each cell type, we selected up to the top 200 cells with the lowest scores as the seed cells for a cell type. In total, 8080 seed cells were identified for 48 normal human lung cell types. We named this collection of seed cells as the LungMAP Human Lung CellRef Seed. To facilitate the use of the CellRef Seed for automated cell type annotation, we normalized gene expression in the seed cells of each datasets using SCTransform, integrated data from different datasets using the RPCA pipeline, and performed UMAP analysis on the integrated data.

We performed a power analysis and determined the minimum cell numbers required for a lung cell type to achieve a power > =0.8. The analysis was performed as follows. First, a Cohen’s *d* effect size was calculated for each cell type using the averaged mean expression and variance of all genes in the cell type of each individual donor when compared to those in all the other cells. We grouped effect size values to the following categories: small ($0.2\le d < 0.5$), medium ($0.5\le d < 0.7$), large ($d\ge 0.7$) and then used the gPower software to calculate a sample size required by each cell type using the following parameters: alpha=0.01, two-tailed *t* test, beta = 0.2, allocation ration = 1. Based on the calculation, a minimum of 50 cells is required to reach the statistical power. 44 out of the 48 human lung cell types meet the criteria; 4 cell types had less than 50 seed cells identified, including chondrocytes (*n* = 6), ILC (*n* = 14), megakaryocyte/platelets (*n* = 29), maDC (*n* = 34).

Using the identified seed cells, we further predicted cell type annotations for all other cells in the 10 datasets collected. Both Seurat’s label transfer and SingleR were applied as described above. The LungMAP Human Lung CellRef (*n* = 347,970 cells) was comprised of the seed cells and the cells with consistent cell type predictions and with kNN-purity scores >=0.6. 157,286 cells that did not pass the criteria were not included, considering the current version of CellRef is guided by a knowledge-based cell directory, those cells may include transitional states or cell types that have not yet defined by the current CellRef.

To facilitate the use of the LungMAP Human Lung CellRef for automated cell type annotation, we normalized gene expression in each donor in the CellRef using SCTransform, integrated data from different donors using the RPCA pipeline, and performed UMAP analysis on the integrated data. During the RPCA integration, we identified “anchors” using the FindIntegrationAnchors function, filtered out “anchors” mapping cells with different cell type predictions, and then used the remaining “anchors” for data integration using the IntegrateData function.

### Construction of the LungMAP Mouse Lung Development CellRef

We constructed a cell type dictionary for mouse lung (Supplementary Data 4) based on our constructed dictionary derived from the LungMAP CellCards. In addition, because of the developmental design of the mouse data, we extended the mouse lung cell type dictionary to include progenitor and transitional cells reported in recent single-cell studies, including *Sox9* + /*Id2*+ distal epithelial cells^23,24^, AT1/AT2 cell, *Foxf1* + /*Kit*+ endothelial progenitor cells^29^, and proliferative mesenchymal progenitor cells^30,31^. We used Seurat to perform SCTransform based data normalization and performed UMAP analysis on the identified LungMAP Mouse Lung Development CellRef Seed and the constructed LungMAP Mouse Lung Development CellRef.

### Automated CellRef annotation of scRNA-seq of normal and diseased human lung

We downloaded and processed published scRNA-seq datasets from normal and disease human lung to demonstrate the utility of automated cell type annotation using the LungMAP human lung CellRefs.

Processed data of scRNA-seq of normal human lung were downloaded from GEO using access numbers GSM5388411, GSM5388412, GSM5388413, GSM4504966, GSM4504967, and GSM4035472. For GSM5388411, GSM5388412, and GSM5388413, cells reported in the original study^33^ (*n* = 6228, 8329, and 7143 cells, respectively) were included. For GSM4504966, GSM4504967, and GSM403547, cells (*n* = 8381, 8034, and 5767, respectively) passing the following criteria were included: at least 500 expressed genes and less than 10% of UMIs mapped to mitochondrial genes.

For scRNA-seq of human lung with lymphangioleiomyomatosis (LAM), we re-processed the data using hg38 reference genome and selected cells (*n* = 12,374) reported in the publication^1^ for the automated CellRef annotation. For scRNA-seq of human lung with idiopathic pulmonary fibrosis (IPF), we downloaded the Seurat object (GSE135893_ILD_annotated_fullsize.rds.gz) from GEO GSE135893. Data (*n* = 57,682 cells) from 19 scRNA-seq samples from 12 IPF lungs were used for the automated CellRef annotation.

Automated CellRef annotation of each testing dataset was performed using the Seurat reference mapping algorithm^5,20^ (FindTransferAnchors and MapQuery functions) using the LungMAP Human Lung CellRef or LungMAP Human Lung CellRef Seed as the reference. FindTransferAnchors was run with the following parameters: normalization.method = ‘SCT’, reference.reduction = ‘pca’, dims = 1:200. MapQuery was run with the following parameters: reference.reduction = ‘pca’, reduction.model = ‘umap’.

### Evaluation of automated CellRef annotation based on prior knowledge

We developed an R script to evaluate cell type annotations predicted by the LungMAP CellRefs based on prior knowledge (CellRef markers, cell type signature genes, and enriched gene sets or pathways). Currently, the functions include: (i) Dotplot visualization of expression levels and frequencies of CellRef marker genes in each of the predicted cell types. Selective and abundant expression of marker genes in their corresponding cell types (*p* value of two-tailed Wilcoxon rank-sum test <0.05, fold change > =1.5 and expression percentage >=0.2) indicate a concordance of cell identities in the predictions and in the CellRef. (ii) Identification of signature genes for each of the predicted cell types. By default, the identification was performed using Seurat’s FindAllMarkers function based on the following criteria: adjusted *p* value of two-tailed Wilcoxon rank-sum test <0.1, pct > =20%, and fold change > =1.5. A sufficient number of signature genes (e.g., >=50 genes) would be expected to define a distinct cell type. (iii) Gene sets functional enrichment analysis (Gene Ontology Biological Process, Pathways) associated with the identified cell type signature genes. Functional enrichment analysis was performed using R package gprofiler2 (v0.2.1). Given scRNA-seq data with automated cell type annotations, the R script can generate the visualizations and evaluations for all predicted cell types at once and compile results into an evaluation report using R markdown.

### Evaluation of automated CellRef cell type annotation using original annotations of published scRNA-seq data

We downloaded the original cell type annotation (45 cell types and states)^33^ of scRNA-seq of adult human lung (GSM5388411, GSM5388412, and GSM5388413) from GEO using GSE178360. The cell type nomenclature and resolutions in the original annotation were different from the CellRef annotations. To match the cell populations in the two annotations for comparison, we performed the following: (i) exclude cell types or states with less than 50 cells, (ii) exclude unique cell types/states that were only present in one annotation, and (iii) merged cell sub-populations that were defined at different granularities between the original and the CellRef annotations. After the processing, 24 matched cell populations were used for the comparison, including AT1, AT2, basal, secretory, SCGB3A2+, ciliated, CAP1, CAP2, arterial/venous/lymphatic/systemic venous endothelial, airway/vascular smooth muscle, alveolar fibroblast, macrophage, dendritic, monocyte, B, plasma, mast/basophil, neutrophil, natural killer, and T cells. For each cell population, we calculated precision, recall, accuracy, F1 score, and Matthews correlation coefficient (MCC) to quantify the consistency between the original and CellRef annotations. These metrics are defined as follows: ${precision}={TP}/({TP}+{FP})$, ${recall}={TP}/({TP}+{FN})$, ${accuracy}=({TP}+{TN})/({TP}+{TN}+{FP}+{FN})$, $F1=2\times ({precision}\times {recall})/({precision}+{recall})$, and ${MCC}=({TP}\times {TN}- {FP}\times {FN})/\sqrt{({TP}+{FP})\times ({TP}+{FN})\times ({TN}+{FP})\times ({TN}+{FN})}$. For a cell population $i$, TP (True Positive) represents the percentage of population $i$ cells predicted by the CellRef that were also identified as population $i$ cells in the original annotation; FP (False Positive) represents the percentage of population $i$ cells predicted by the CellRef that were not in the population $i$ of the original annotation; TN (True Negative) represents the percentage of cells not annotated as population $i$ by the CellRef and were also not in the population $i$ of the original annotation, FN (False Negative) represents the percentage of cells not annotated as population $i$ by the CellRef but were in the population $i$ of the original annotation.

### Evaluation of CellRef cell type annotations using 10-fold cross validation

We performed 10-fold cross validation for both LungMAP human and mouse CellRefs. In each case, we randomly divided the data into 10 equal parts using the KFold function in the R package rBayesianOptimization (v1.2.0). The partitions were performed for each cell type so that each of the 10 data parts contains similar cell type distributions. Cell types with more than 500 cells were used in the cross validation analysis so that each data part contained more than 50 cells of each cell type. The design is based on our power analysis which determined that 50 is the minimum cell numbers required for a lung cell type to achieve a power > =0.8. For each round of validation, we used 9 parts as training data to predict cell types of the remaining part (testing data) using the Seurat reference mapping algorithm. In total, 10 runs of the training and testing were performed. At each run, a different data part was used as the testing data and the prediction performance was measured by calculating an F1 score and a Matthews correlation coefficient (MCC) for each cell type in the testing data. We reported the distributions of the F1 and MCC scores of all cell types in a cross validation analysis and considered the median scores as the overall performance.

### Cell type stability analysis

We calculated cell type stability metrics (SCCAF^39^, Reassign^18^, TFIDF10^18^) using scTriangulate^18^ (v0.12.0, https://github.com/frankligy/scTriangulate). Single-cell clustering assessment framework (SCCAF) randomly splits data into a training and a testing set, considers all features in the training set to build a classifier to predict cell labels of a testing set and compare with the reference annotations in the CellRefs or HLCA. The reassign scores measure cell-to-cluster re-assignment accuracy by measuring the fraction of cells in each cluster that can be re-classified to its own centroid. TFIDF10 scores measure cluster marker gene specificity by the strength of the 10th most exclusively expressed feature in a cluster. The HLCA core reference (v1.0) was downloaded as an h5ad file from the cellxgene (https://cellxgene.cziscience.com/). We applied scTriangulate to the LungMAP Human and Mouse Lung CellRefs and the HLCA, separately, calculated the SCCAF, Reassign, and TFIDF10 scores for each cell type in the human lung CellRef, mouse lung CellRef, and HLCA. LogNormalize gene expression data was used in the calculations. For CellRef, we used the annotations of 48 human lung and 40 mouse lung cell types. For HLCA, we used the ann_finest_level original annotation of 58 cell types^37^.

We also assessed the stability of human lung CellRef and HLCA by projecting their annotations to the previously-reported human lung scRNA-seq atlas^15^, which was downloaded as an h5ad file from cellxgene (https://cellxgene.cziscience.com/) and supplied as an input to Azimuth instances for the cell type annotation using the LungMAP Human Lung CellRef Seed (https://app.lungmap.net/app/azimuth-human-lung-cellref-seed) and the integrated HLCA (https://app.azimuth.hubmapconsortium.org/app/human-lung-v2). The mapped annotations from the CellRef and HLCA were analyzed and visualized (UMAP) in scTriangulate^18^ using default program options.

### Assessment of cell type identity mapping between CellRef and HLCA using pseudo-bulk-based correlation analysis

To assess the cell identity and mapping of cell types in the human lung CellRef and HLCA, we first created a pseudo-bulk gene expression profile for each cell type by averaging the expression of each gene of all cells in the given cell type. Then Seurat’s FindVariableFeatures was used to find the top 2000 highly variable genes (HVGs) among the pseudo-bulk profiles of the CellRef, denote HVG1, and the HVGs among the pseudo-bulk profiles of the HLCA, denote HVG2. We took the union of HVG1 and HVG2 and kept the genes that are present in both references, resulting in 2501 HVGs. We performed zscore scaling of the expression of 2501 genes among the CellRef and the HLCA pseudo-bulk profiles, separately. Pearson’s correlations among all the pseudo-bulk profiles of CellRef and HLCA were calculated using the scaled expression of 2501 HVGs. Hierarchical clustering analysis was performed using R package pheatmap (v1.0.12) using the correlation matrix as input.

### Marker-based assessment of cell type accuracy using receiver operator characteristics (ROC) analysis

In this analysis, we used area under the ROC curve (AUC) to assess the accuracy of each cell type in a single-cell reference based on its consistency with the expression of cell type selective marker genes. Let $X$ be the set of all cells in the reference, ${X}_{i}\,{{\subseteq }}{X}$ be the cells of cell type $i$ in the reference, and ${Y}_{i}$ be the set of marker genes of the cell type $i$. For each marker gene $y\in {Y}_{i}$, we generated a ranking of $X$ according to the decreasing order of the zscore-transformed expression of $y$ in $X$. Then we generated a global ranking of $X$ by merging all the rankings by ${Y}_{i}$ using the aggregateRanks function in the R package RobustRankAggreg (v1.2.1). The AUC score for the cell type $i$ was calculated by comparing this global ranking with the cell type annotation in the reference, i.e., all cells in ${X}_{i}$ were considered as positive instance; otherwise, negative. The AUC scores were calculated using the roc function in the pROC (v1.18.0) package with default parameters.

The human lung CellRef cell type selective markers were generated by including dictionary marker genes (Supplementary Data 2) and top selectively expressed markers for each cell type. Up to 10 marker genes were selected for each cell type using the following criteria. For each cell type in the human lung CellRef, we identified its specific differentially expressed genes (DEGs) in CellRef and CellRef Seed using the following criteria: adjusted *p* value of two-tailed Wilcoxon rank-sum test <0.1, expression percentage >= 30%, fold change of average expression >=1.5, and recall >10%. Top-ranked cell type specific DEGs (ranked by fold change in average expression) were combined with the known markers genes to form the CellRef cell type selective marker gene list (Supplementary Data 5, up to 10 genes for each of the 48 CellRef human lung cell types). DE tests were performed using the FindAllMarkers function in Seurat (v4.1.0) with the following parameters: test.use = ”wilcox”, assay = ”RNA”, only.pos=T. The recall of a gene expression in a cell type was calculated as the number of cells in the cell type with positive expression (>0) of the gene divided by the total number of cells with positive expression of the gene. Using the same approach, the mouse lung CellRef cell type selective marker genes were selected (Supplementary Data 6, up to 10 marker genes per cell type) and used for the AUC based assessment of mouse lung CellRef. The human and mouse CellRef markers are also openly accessible at LGEA CellRef (https://research.cchmc.org/pbge/lunggens/CellRef/LungMapCellRef.html). The HLCA predicted markers genes were downloaded from Sikkema et al.^37^, which contains up to 10 genes for each of the 58 HLCA cell types.

### Assessment of single-cell data integration using the Local Inverse Simpson’s Index (LISI) metrics

We assessed the data integration in the LungMAP CellRefs and HLCA using the LISI metrics (https://github.com/immunogenomics/LISI, v1.0), including integration LISI (iLISI) and cell-type LISI (cLISI). Given a single-cell data integration (CellRef or HLCA), an iLISI score was calculated for each cell in each cell type using the compute_lisi function in LISI package with the following parameters: the UMAP coordinates of all cells in the selected cell type and the batch information (donor or data sample) of all cells in the selected cell type. We normalized the iLISI score of each cell using the total number of batches in the cell type of the cell. Given an integrated single-cell data, a cLISI score was calculated for each cell using the compute_lisi function in LISI package with the following parameters: the UMAP coordinates and the cell type information of all cells in the integration. For the CellRef, we used the annotation of 48 cell types for the cLISI calculation. For the HLCA, we used the ann_finest_level annotation of 58 cell types for the cLISI calculation.

### Statistical analysis

Statistical analyses of differences in the area under the receiver operating characteristics curves (AUCs), cell type stability scores, and data integration scores were performed in R (v 4.1.0) using Welch’s *t* test (two-tailed, unequal variance). Multiple testing correction was performed using Bonferroni correction. The results are expressed as violin plots or box plots representing 25%, 50%, and 75% quantiles, with mean ± SD or SEM error bars, as noted in individual figure legends. Differential expression analysis of single-cell gene expression was performed in the R package Seurat (v 4.1.0) using two-tailed Wilcoxon rank-sum test.

### Reporting summary

Further information on research design is available in the Nature Portfolio Reporting Summary linked to this article.

## Supplementary information

Supplementary Information

Description of additional supplementary files

Supplementary Data 1

Supplementary Data 2

Supplementary Data 3

Supplementary Data 4

Supplementary Data 5

Supplementary Data 6

Reporting Summary

## Data Availability

Published single cell/nucleus RNA-seq of human lung used in the human lung CellRef are available in the Gene Expression Omnibus under accession codes “GSE135893”, “GSE136831”, “GSE122960”, “GSE134174”, “GSE161382”, “GSE171524”, in the European Genome-phenome Archive under accession code “EGAS00001004082 [https://ega-archive.org/studies/EGAS00001004082]”, and in the Synapse.org under accession code “syn21041850 [https://www.synapse.org/#!Synapse:syn21041850]”. The LungMAP CCHMC and UPenn data used in this study are available in the LungMAP.net under accession code “LMEX0000004396”. Drop-seq of mouse lung data used in the mouse lung CellRef are available in the Gene Expression Omnibus under accession code “GSE122332” and in the LungMAP.net under accession code “LMEX0000004397”. Published single cell RNA-seq of human lung data used in the evaluation analysis are available in the Gene Expression Omnibus under accession codes “GSE178362”, “GSE135893”, “GSE135851”, and “GSE149563”. The HLCA core reference (version 1.0) used in the benchmarking analysis is available at FASTGenomics under accession code “dataset-427f1eee6dd44f50bae1ab13f0f3c6a9 [https://beta.fastgenomics.org/datasets/detail-dataset-427f1eee6dd44f50bae1ab13f0f3c6a9]”. Web interfaces for the human and mouse lung CellRefs are available at Lung Gene Expression Analysis (LGEA) web portal (https://research.cchmc.org/pbge/lunggens/CellRef/LungMapCellRef.html) and LungMAP.net (https://lungmap.net/cell-cards/, “CellRef scRNA-seq” tab). All other data supporting the findings of this study are available within the article and its supplementary files. Any additional requests for information can be directed to, and will be fulfilled by, the lead contact. Source data are provided with this paper in ‘SourceData.zip’.

## Source data

Source Data

## Code availability

The code of LungMAP CellRef construction pipeline and the code to reproduce the analyses are available on GitHub: https://github.com/xu-lab/CellRef^19^.
