# Supplementary material for: Guided construction of single cell reference for human and mouse lung
Source: Nat Commun. 2023 Jul 29;14:4566. doi: 10.1038/s41467-023-40173-5 (PMC10387117; doi:10.1038/s41467-023-40173-5)
Supplement: Supplementary file 1 — Supplementary Information [file 41467_2023_40173_MOESM1_ESM.pdf]

Guided construction of single cell reference for human and mouse lung

Guo et al.

Supplementary Figures

Supplementary Figure 1. Data integration and identification of the LungMAP Human Lung CellRef Seed. (**A**) Integration of cells from individual donors of seven single cell RNA-seq datasets. (**B**) Comparison of data integration using three single cell batch correction methods (MNN[1], Harmony[2], RPCA[3]) included in our construction pipeline (see Methods). Reassign[4] and SCCAF[5] were used for cluster stability assessment (details see Method section). Gene expression matrix was Log normalized using Seurat, and Leiden[6] was used for clustering (with default resolution=0.8). Data distributions are visualized using violin plots (Harmony: n=42 cell clusters; MNN: n=42 cell clusters; RPCA: n=35 cell clusters). The black dots and error bars represent mean±SEM. p values represent significance of difference tested using two-tailed unpaired Welch's *t* test and adjusted for multiple testing using Bonferroni correction. MNN-based data integration showed the best performance among the three; we therefore set MNN as default in our pipeline and used it for the results in Supplementary Figure 1C-1D. (**C**) Cells colored by regions of tissue samples. (**D**) Cell clusters identified using the Leiden algorithm. (**E**) The identified seed cells (dots with non-grey colors) for each of the 48 cell types in the dictionary. Chon.: chondrocyte; Meso.: mesothelial; Megaka.: megakaryocyte. Please see Figure 2 for definitions of cell type abbreviations.

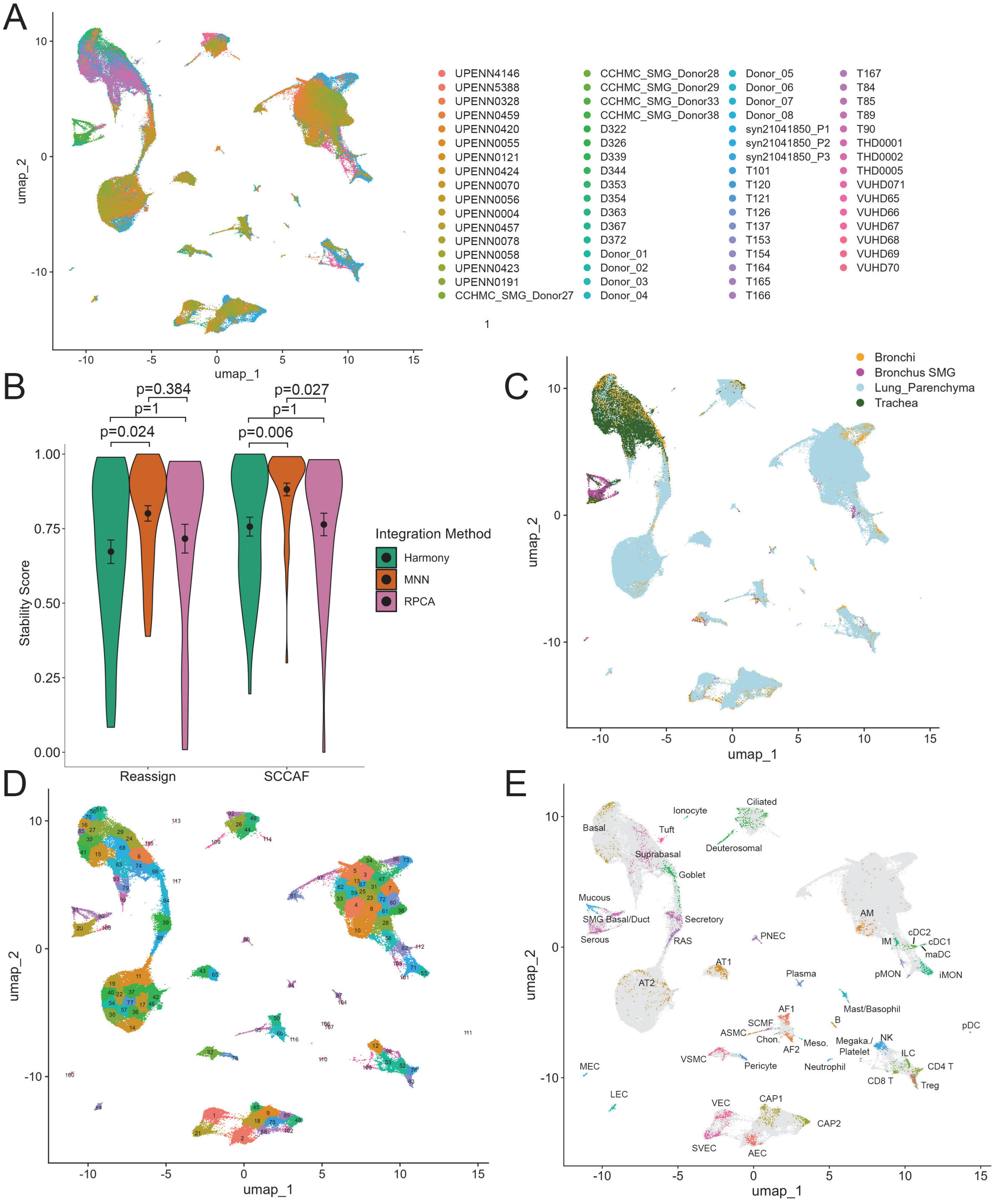

Supplementary Figure 2. Mapping individual single cell/nucleus (sc/sn) RNA-seq datasets of normal human lung to the LungMAP Human Lung CellRef Seed. UMAP plots showed the LungMAP Human Lung CellRef cells from each dataset colored by (**A**) cell type annotations predicted using the CellRef Seed as reference using both the Seurat's label transfer method and SingleR and (**B**) cell type annotations in the original studies. Please see Figure 2 for definitions of cell type abbreviations in (A). No UMAP plots of three datasets (CCHMC LungMAP, UPenn LungMAP, and GSE122960) in B as they were either unpublished data or no original annotations available.

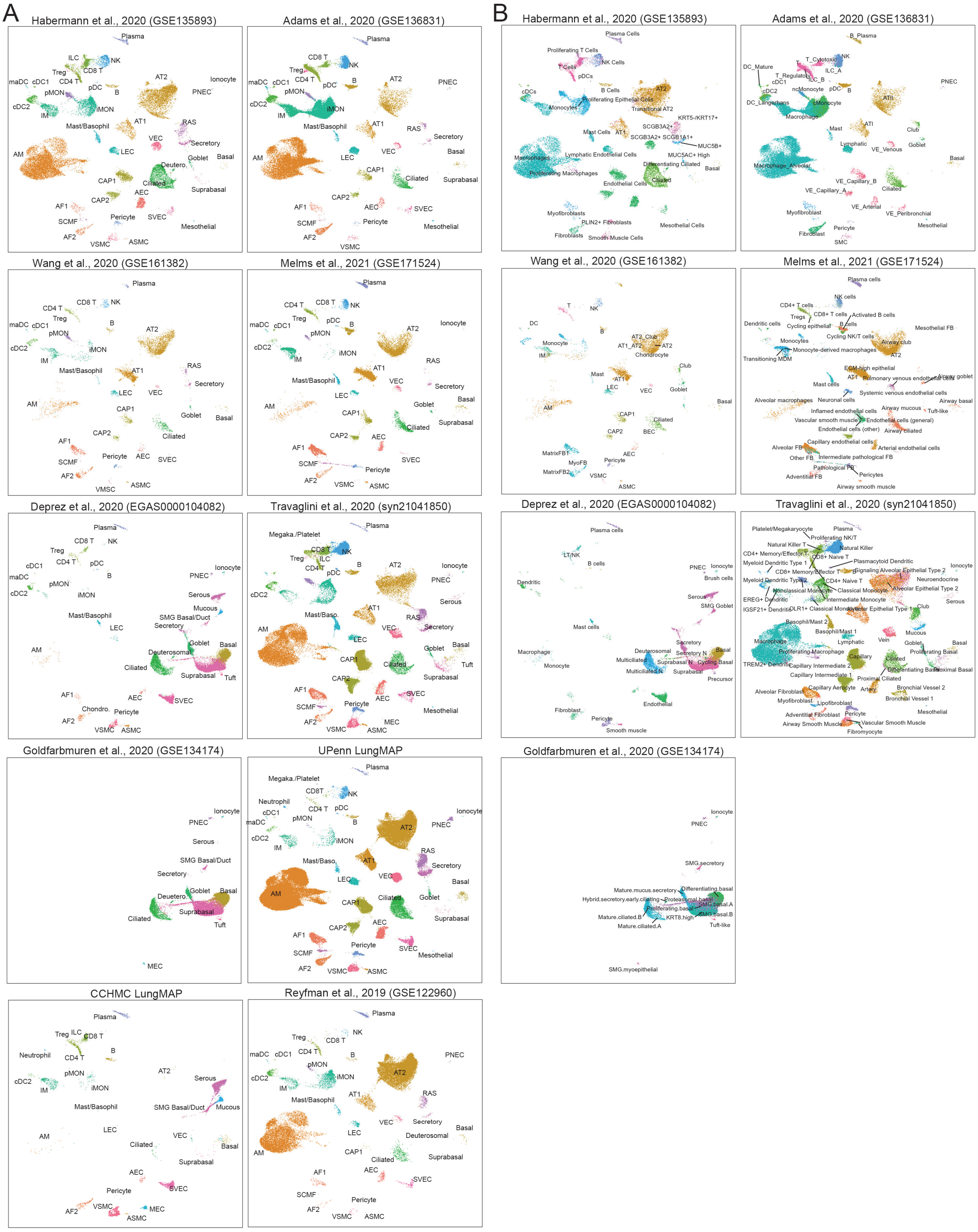

Supplementary Figure 3. Cellular composition and cell type marker gene expression in the LungMAP Human Lung CellRef. (**A**) Dotplot visualization of expression of cell type marker genes from the LungMAP CellCards in each cell type of the LungMAP Human Lung CellRef. Gene expression was measured by unique molecular identifier (UMI) and normalized using Seurat's LogNormalize function. (**B**) The number of cells in each cell type in the CellRef. Megaka.: megakaryocyte. Please see Figure 2 for definitions of cell type abbreviations.

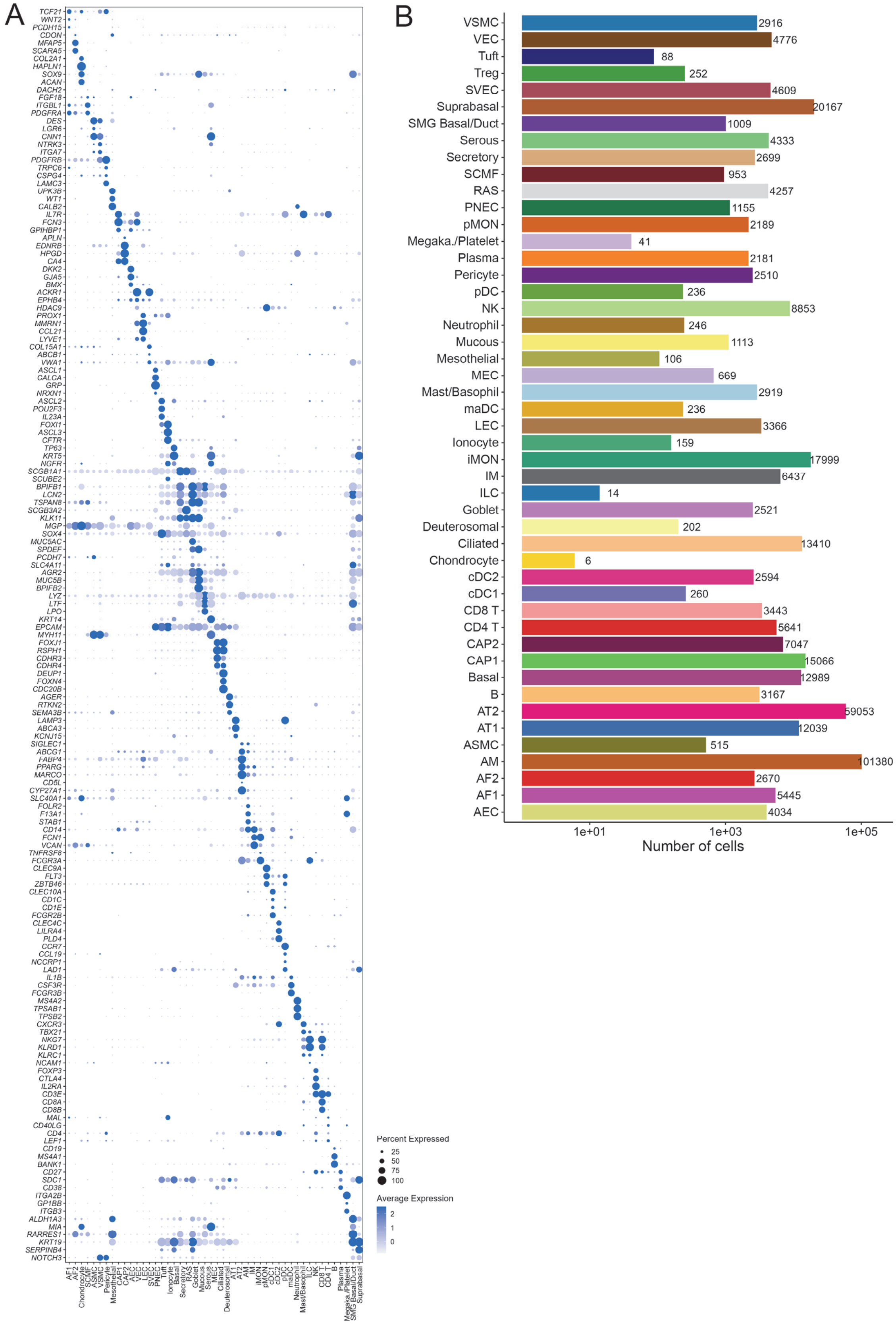

Supplementary Figure 4. Data integration and identification of the LungMAP Mouse Lung Development CellRef Seed. (**A**) UMAP visualization of integrated data from 17 Drop-seq samples from eight time points of mouse lung development. (**B**) Cells colored by developmental time points. (**C**) Cell clusters identified using the Leiden algorithm. (**D**) The identified seed cells for each of the 40 cell types in developmental mouse lung. (**E**) Dynamic cell composition distribution of the 40 cell types in the LungMAP Mouse Lung Development CellRef in each of the eight development time points. Please see Figure 3 for definitions of cell type abbreviations.

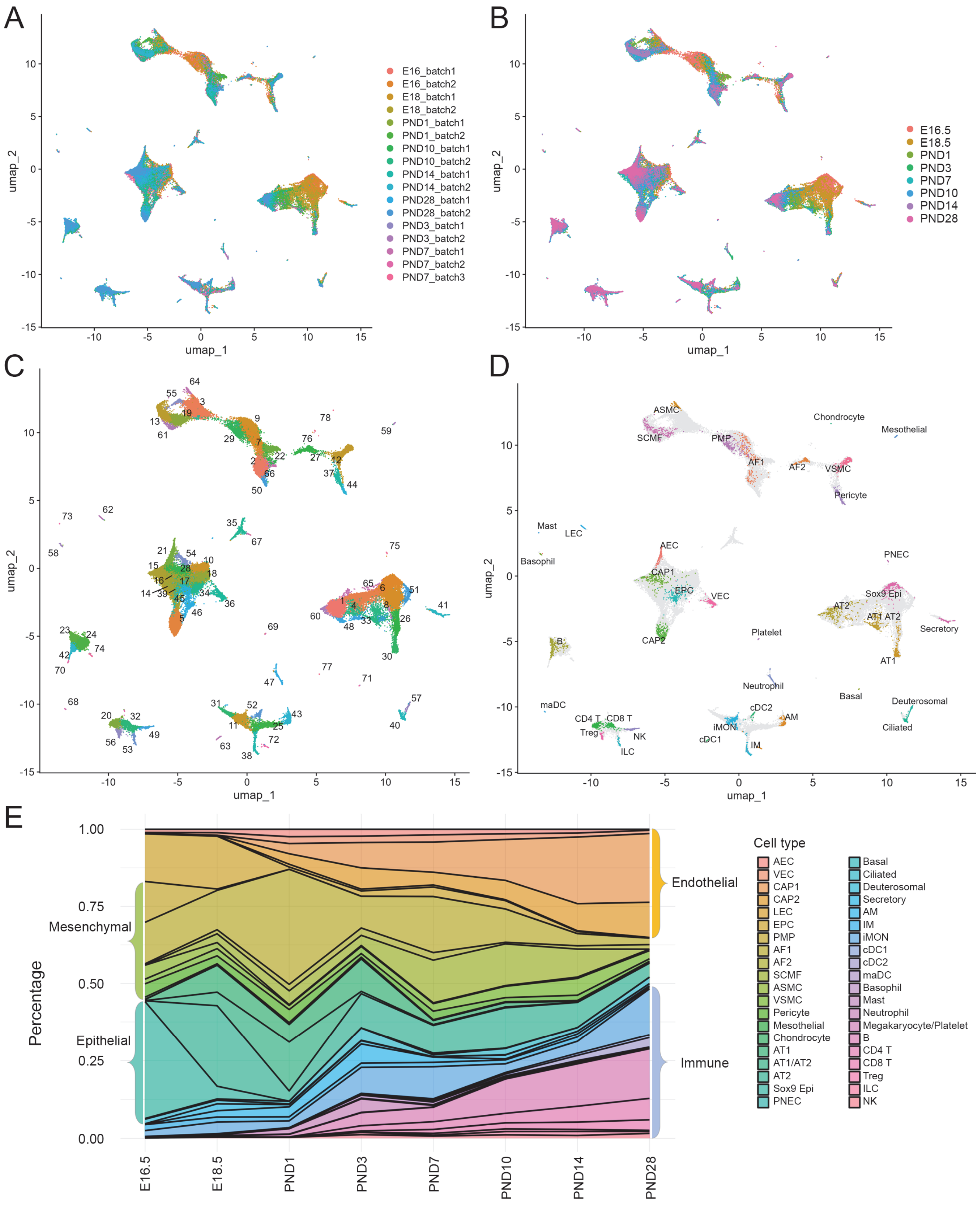

Supplementary Figure 5. UMAP projection comparison of CellRef cells in male vs female, 3' vs 5' samples, and scRNA vs. snRNA profiling. (**A**) UMAP visualizations of CellRef cells by 10X Single Cell 3' or 5'. Left: UMAP of CellRef data from 3' single cell RNA-seq (scRNA-seq) datasets (GSE122960, GSE136831, UPenn LungMAP). Right: UMAP of CellRef data from 5' scRNA-seq datasets (GSE135893). (**B**) UMAP visualizations of CellRef cells/nuclei in male vs female. CellRef data from all the integrated scRNA and single nucleus RNA-seq (snRNA-seq) datasets (n=10) were used in both left and right panels. (**C**) UMAP visualizations of CellRef cells/nuclei in scRNA vs snRNA sequencing. Left: UMAP of CellRef data from scRNA-seq (3' and 5') datasets (GSE122960, GSE135893, GSE136831, UPenn LungMAP). Right: UMAP of CellRef data from snRNA-seq datasets (GSE161382 and GSE171524). Please see Figure 2 for definitions of cell type abbreviations.

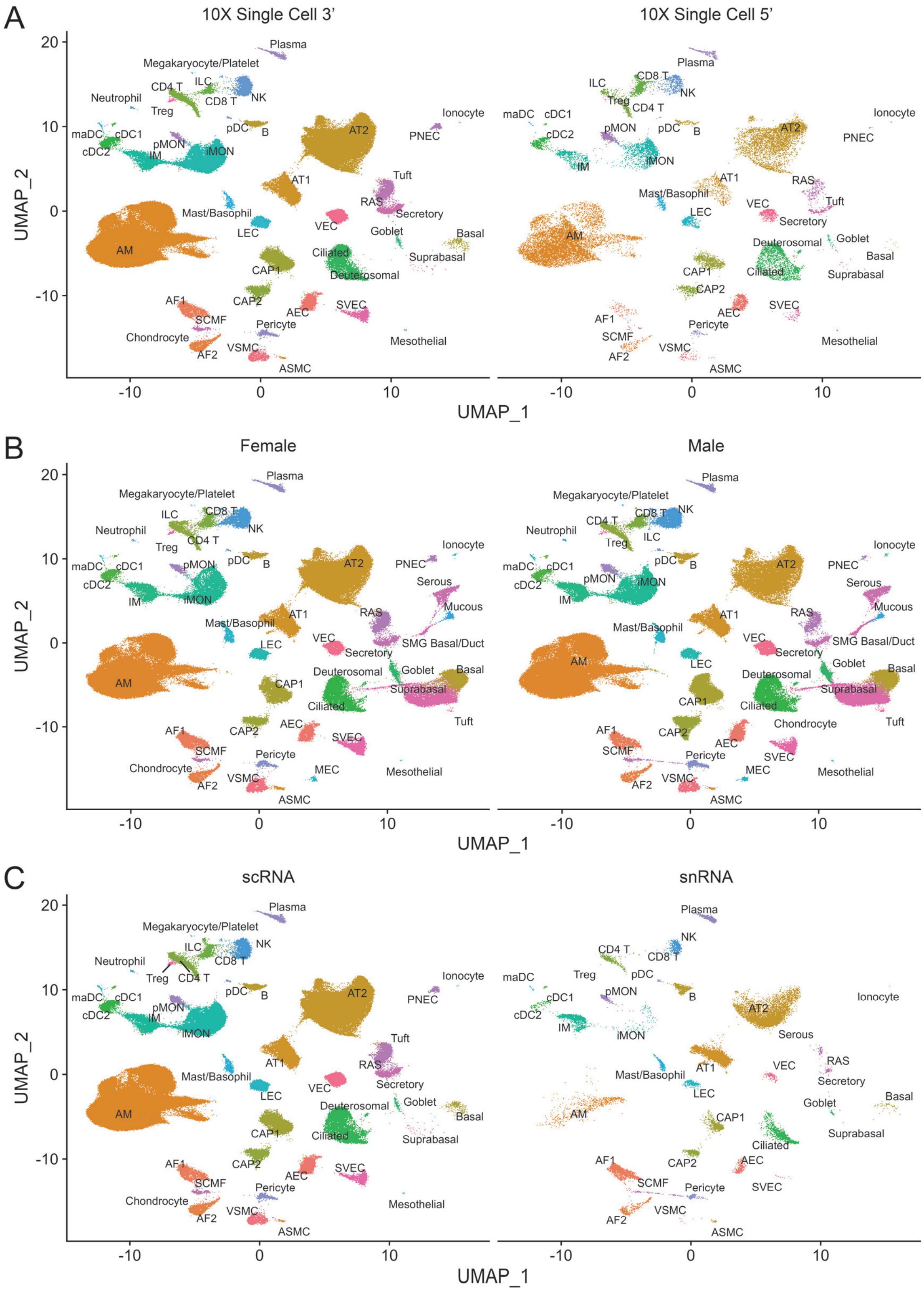

Supplementary Figure 6. Interactive exploration of LungMAP CellRefs using scViewer-lite. (**A**) Using scViewer-lite to query for the expression pattern of a single gene. Left panel shows user interfaces to enter the query. Right panels show UMAP plots of the expression of the query gene and the cell types in the LungMAP Human Lung CellRef Seed. (**B**) Side-by-side UMAP visualizations of expression of two genes of interests using scViewer-lite.

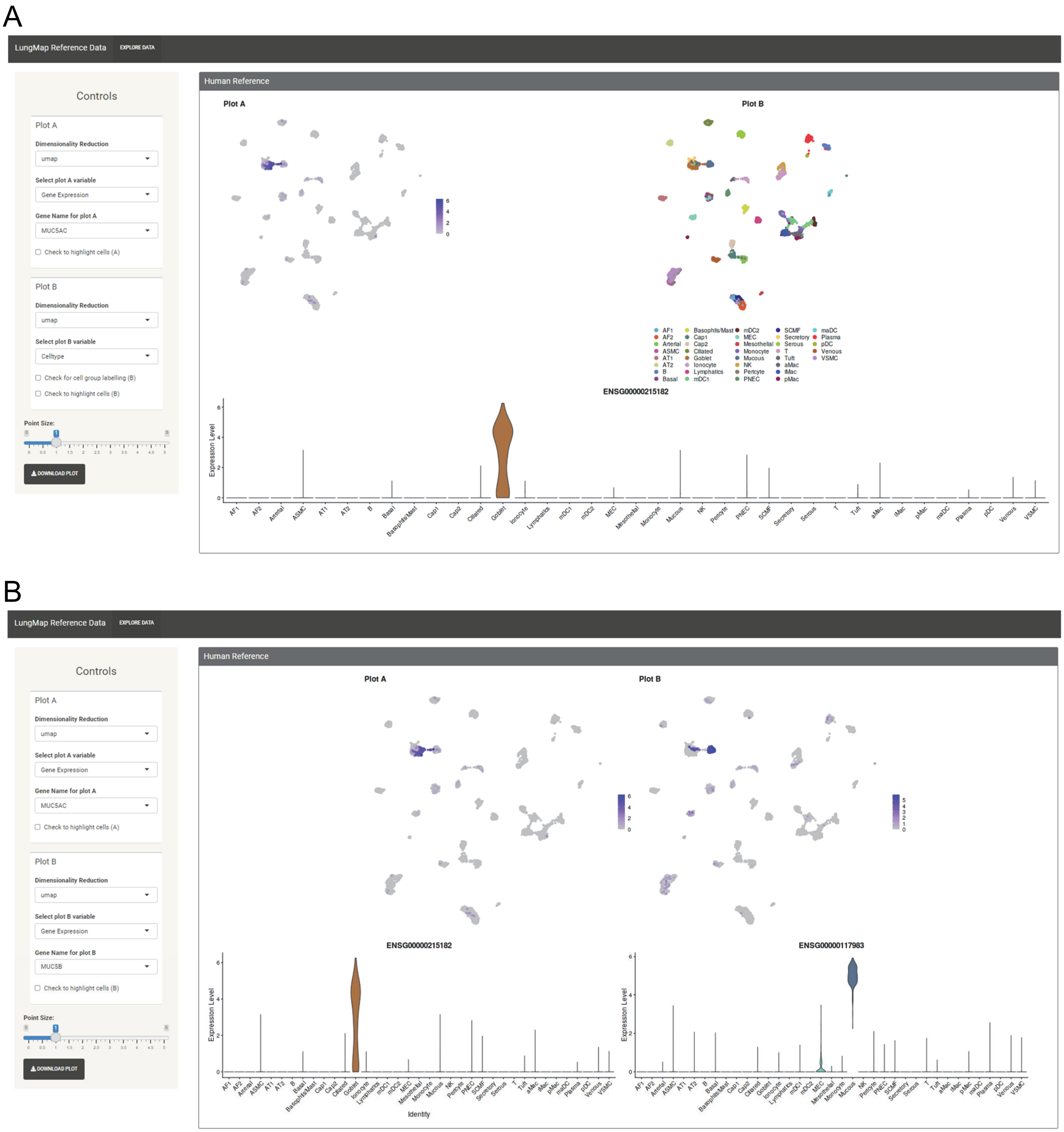

Supplementary Figure 7. Evaluation of automated cell type annotations for scRNA-seq of normal human lung using the LungMAP CellRefs. (A) UMAP of cells from scRNA-seq of a 2-month-old normal human lung (GSM4504966 and GSM4504967). (**B**) Dotplot visualization of expression of CellRef marker genes in the predicted cell types in A. (**C**) UMAP of cells from scRNA-seq of a 31-year-old normal human lung (GSM4035472). (**D**) Dotplot visualization of expression of CellRef marker genes in the predicted cell types in C. In (A) and (C), cells were colored by cell type annotations predicted using the "LungMAP Human Lung CellRef Seed" as reference. Basal and suprabasal cells were combined in the automated annotation. Cells passed selection thresholds of (i) prediction scores greater than or equal to cutoff (1 standard deviation lower than the mean) and (ii) the number of cells per cell type ≥ 5 cells were shown in UMAP and Dot plots. (B) and (D) showed expression of CellRef cell type markers that were differentially expressed (p value <0.05, fold change>=1.5 and expression percentage >=0.2) in their corresponding cell type predictions in (A) and (C), respectively. Seurat v4 FindMarkers function with two-tailed Wilcoxon rank sum test was used for differential expression test. Megaka.: megakaryocyte. Please see Figure 2 for definitions of cell type abbreviations.

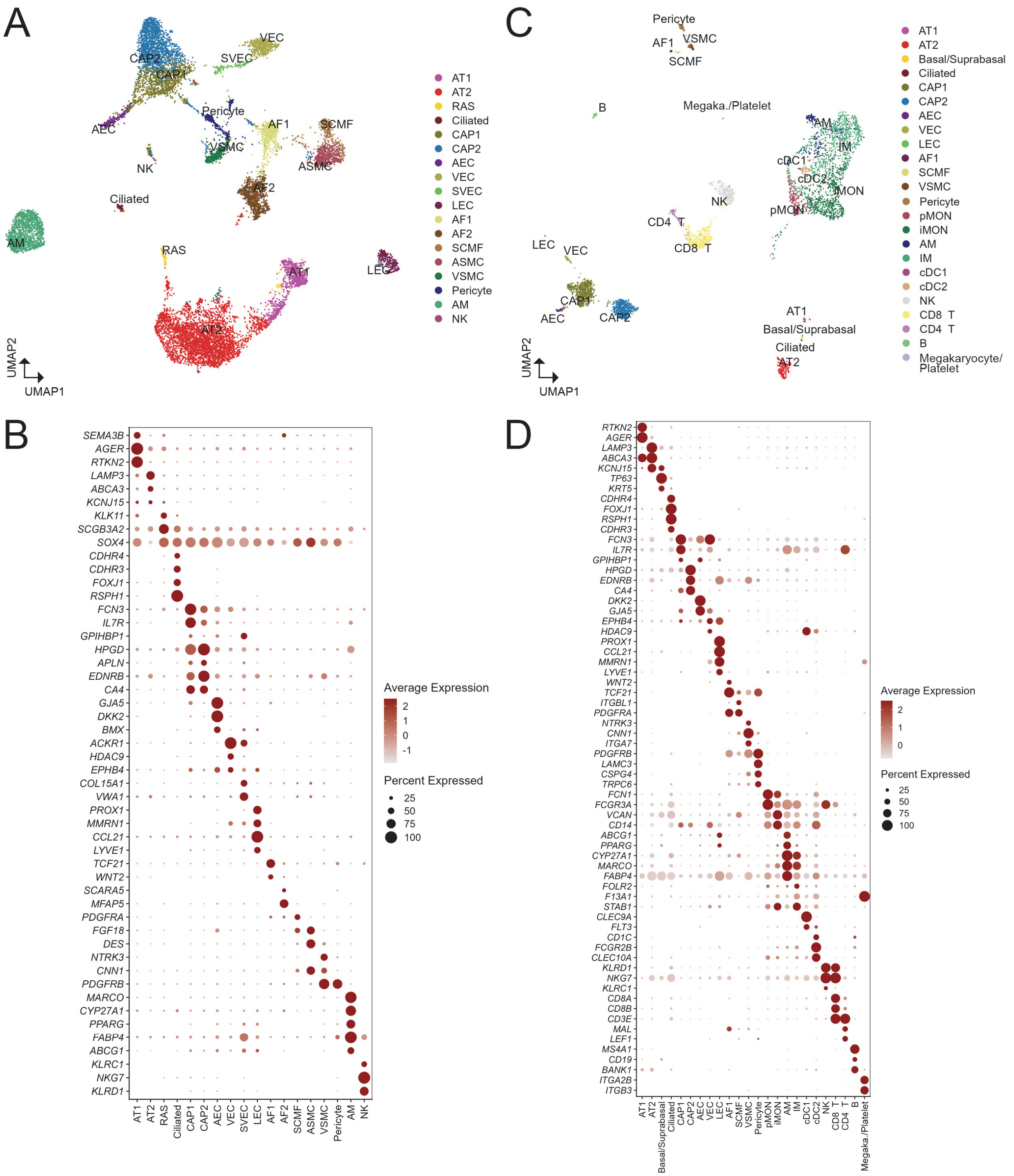

Supplementary Figure 8. Comparison of CellRef annotation and original annotation of published scRNA-seq data of normal human lung from GSE178360. Processed data and published cell type annotation of scRNA-seq of normal adult human lung (n=3 lungs, GSM5388411/12/13) were downloaded from the data series GSE178360 in Gene Expression Omnibus**.** CellRef annotations were predicted by the Seurat reference mapping algorithm using the LungMAP Human Lung CellRef seed as the reference. A processing was performed to match cell populations in the original and the CellRef annotations for comparison (Methods). The consistency of each of the corresponding cell populations (n=24 cell populations) between the two annotations was measured using F1 score, precision, recall, accuracy, and Matthews correlation coefficient (MCC) as defined in the Methods section. Box center lines, bounds of the box, and whiskers indicate medians, first and third quartiles, and minimum and maximum values within 1.5×IQR (interquartile range) of the box limits, respectively.

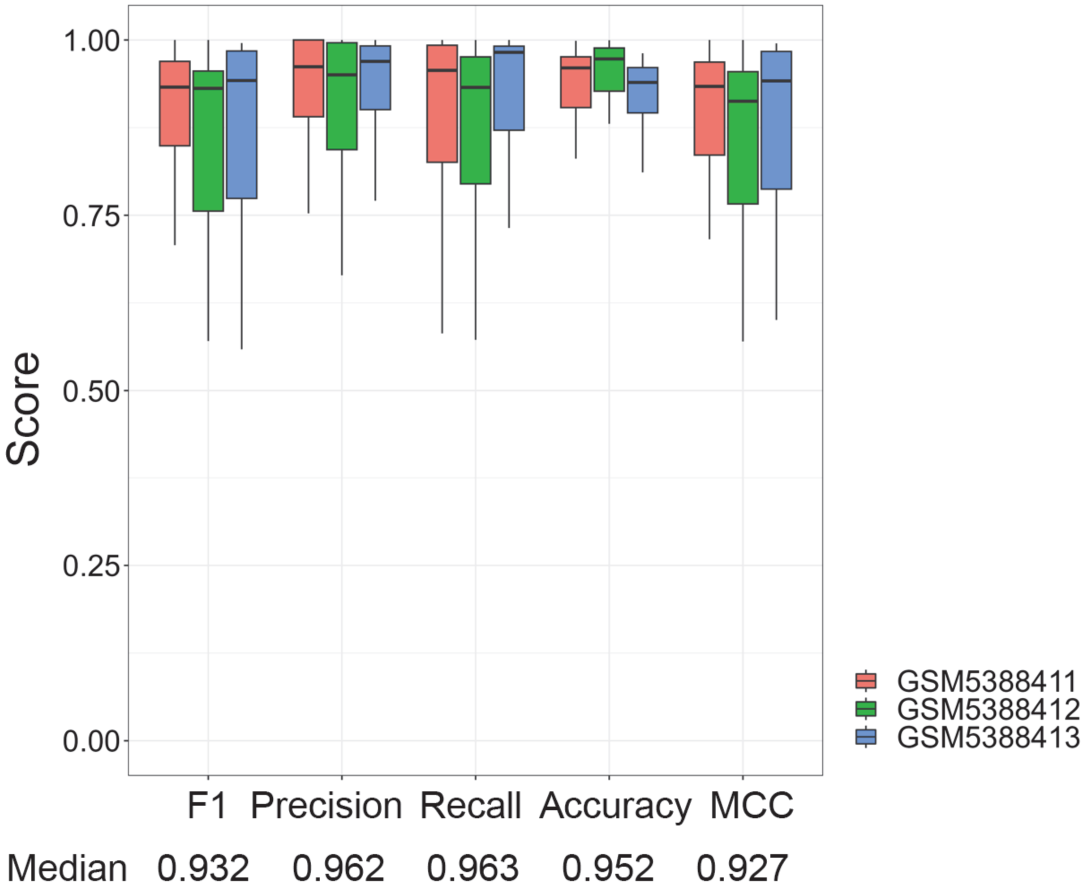

Supplementary Figure 9. Cross validation of LungMAP human and mouse lung CellRefs. For either the human (left) or mouse (right) lung CellRef, we performed a 10-fold cross validation analysis (Methods) and measured the performance of each cell type using F1 score and Matthews Correlation Coefficient (MCC). Cell types with more than 500 cells were included in the cross-validation analysis (n=36 human lung cell types; n=26 mouse lung cell type). Red and green horizontal lines represent median F1 and MCC scores, respectively. Box center lines, bounds of the box, and whiskers indicate medians, first and third quartiles, and minimum and maximum values within 1.5×IQR (interquartile range) of the box limits, respectively.

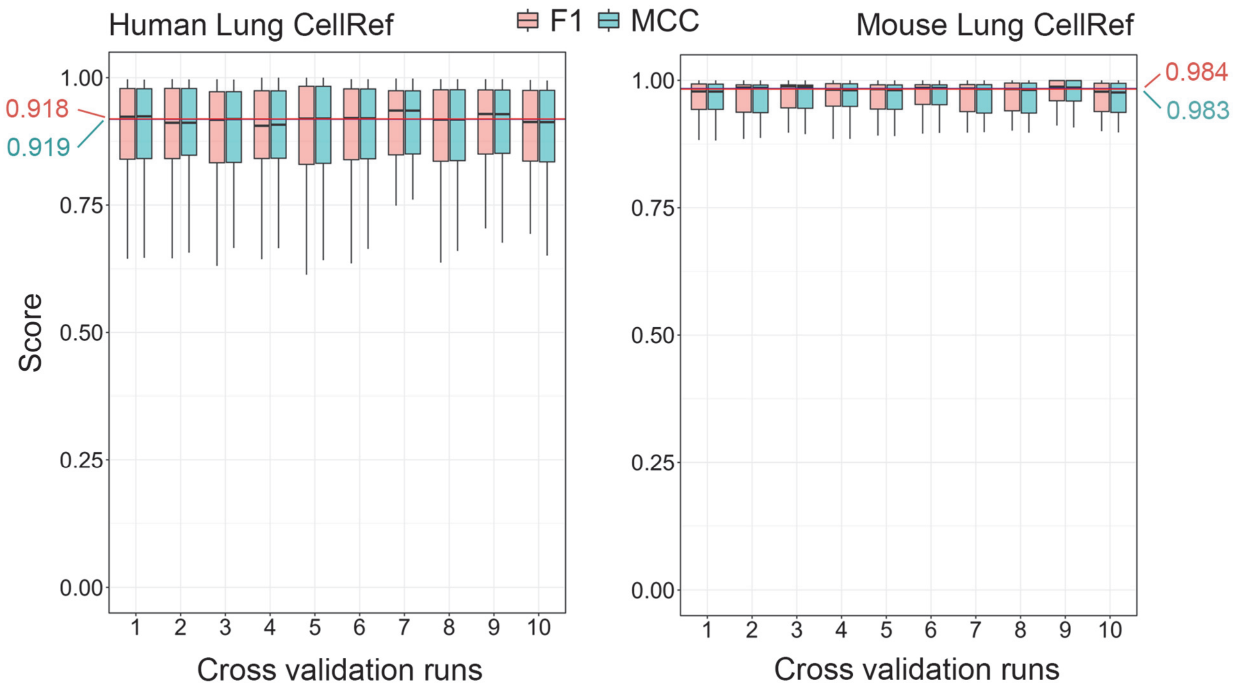

Supplementary Figure 10. Cross comparison of cell types in the LungMAP Human Lung CellRef and the integrated version Human Lung Cell Atlas (HLCA) based on expression of HLCA cell type markers. (**A**) The summary of one-to-one cell type mapping between the two references. (**B**) and (**C**) showed examples of good and unconfident (marked with "?") cell type mappings, respectively. (**B**) Dotplot shows the expression of HLCA adventitial fibroblasts markers in the LungMAP Human Lung CellRef Seed (left) and the LungMAP Human Lung CellRef (right). All marker genes were consistently and selectively expressed in the AF2 cell in the CellRef, suggesting a good mapping of HLCA adventitial fibroblasts and CellRef AF2. (**C**) Dotplot shows the expression of HLCA subpleural fibroblasts markers in the CellRef Seed (left) and the CellRef (right). No confident cell type mapping was reached. Megaka.: megakaryocyte.

A

LungMAP Human Lung CellRef	HLCA
Alveolar fibroblast 2 (AF2)	Adventitial fibroblasts
Alveolar fibroblast 1 (AF1)	Alveolar fibroblasts
Alveolar type 1 cell (AT1)	AT1
Alveolar type 2 cell (AT2)	AT2
B cell (B)	B cells
Basal cell (Basal)	Basal resting
CD4+ T cell (CD4 T)	CD4 T cells
CD8+ T cell (CD8 T)	CD8 T cells
Inflammatory monocyte (iMON)	Classical monocytes
Goblet cell (Goblet) / Secretory cell (Secretory)	Club (non-nasal)
Classical DC subset 1 (cDC1)	DC1
Classical DC subset 2 (cDC2)	DC2
Deuterosomal cell (Deuterosomal)	Deuterosomal
Capillary 2 cell (CAP2)	EC aerocyte capillary
Arterial endothelial cell (AEC)	EC arterial
Capillary 1 cell (CAP1)	EC general capillary
Venous endothelial cell (VEC)	EC venous pulmonary
Systemic venous endothelial cell (SVEC)	EC venous systemic
Goblet cell (Goblet)	Goblet (bronchial)
Goblet cell (Goblet)	Goblet (subsegmental)
Ionocyte cell (Ionocyte)	Ionocyte
Lymphatic endothelial cell (LEC)	Lymphatic EC differentiating
Lymphatic endothelial cell (LEC)	Lymphatic EC mature
Mast/Basophil (Mast/Basophil)	Mast cells
Mesothelial cell (Mesothelial)	Mesothelium
Mature DC subset (maDC)	Migratory DCs
Ciliated cell (Ciliated)	Multiciliated (non-nasal)
Secondary crest myofibroblast (SCMF)	Myofibroblasts
Pulmonary neuroendocrine cell (PNEC)	Neuroendocrine
Natural killer cell (NK)	NK cells
Patrolling monocyte (pMON)	Non-classical monocytes
Pericyte (Pericyte)	Pericytes
Plasma cell (Plasma)	Plasma cells
Plasmacytoid DC (pDC)	Plasmacytoid DCs
SMG Basal/Duct cell (SMG Basal/Duct)	SMG duct
Mucous cell (Mucous)	SMG mucous
Serous cell (Serous)	SMG serous (bronchial)
Vascular smooth muscle cell (VSMC)	Smooth muscle
Suprabasal cell (Suprabasal)	Suprabasal
Respiratory airway secretory cell (RAS)	Transitional Club-AT2
Tuft cell (Tuft)	Tuft
Interstitial macrophage (IM)	Interstitial Mφ perivascular
Alveolar macrophage (AM)	Alveolar macrophages
Alveolar macrophage (AM)	Alveolar Mφ CCL3+
Airway smooth muscle cell (ASMC) ?	Fibromyocytes
Mesothelial cell (Mesothelial) ?	Subpleural fibroblasts
Secondary crest myofibroblast (SCMF) ?	Peribronchial fibroblasts
	Alveolar Mφ MT-positive
	Monocyte-derived Mφ
	AT2 proliferating
	Lymphatic EC proliferating
	Alveolar Mφ proliferating
	T cells proliferating
	SM activated stress response
	Club (nasal)
	Multiciliated (nasal)
	SMG serous (nasal)
	Goblet (nasal)
Myoepithelial cell (MEC)	
Neutrophil	
Megakaryocyte/Platelet	
T regulatory cell (Treg)	
Innate lymphoid cell (ILC)	
Chondrocyte	

B

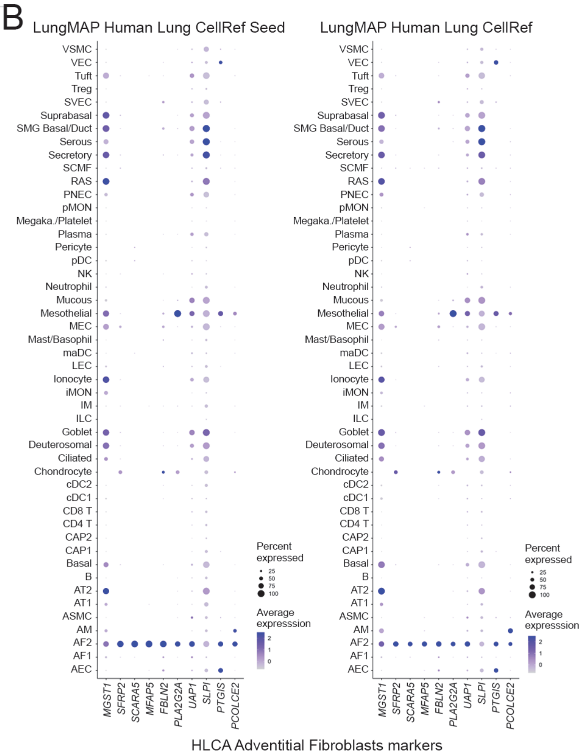

C

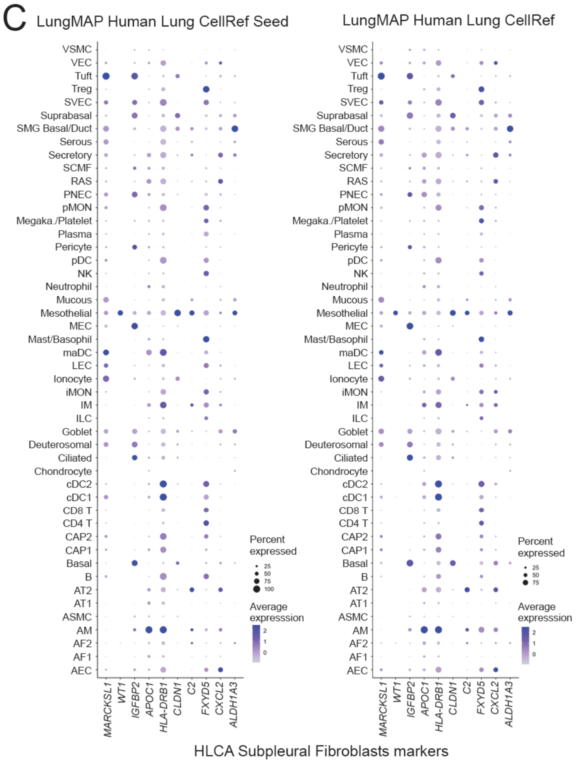

Supplementary Figure 11. Assessment of cell type stability and data integration in the LungMAP Human Lung CellRef and HLCA. (**A**) Assessment of stability of CellRef cell types (n=48) and HLCA cell types (n=58) based on three metrics (Reassign, SCCAF[5], and TFIDF10) calculated using scTriangulate[4] with default parameters. Stability scores were calculated for each cell type in each reference. Data distributions are visualized using violin plots. The black dots and error bars represent mean±SEM. (**B**) Assessment of data integration in the CellRef and HLCA using the Local Inverse Simpson's Index (LISI) metrics[2], including integration LISI (iLISI, assessing batch mixing) and cell-type LISI (cLISI, assessing cell type separation). The "subjectID" and "sample" in the HLCA meta data were used as the "DonorID" and "DataID", respectively. Both the iLISI and cLISI scores were calculated for each cell in each reference. Boxplot represents 25%, 50%, and 75% quantiles. The black dots and error bars represent mean±SD for iLISI scores (left panel, n=932,854 cells) and mean+SD for cLISI scores (right panel, n=932,854 cells). *P* values in (A) and (B) represent significance of difference tested using two-tailed unpaired Welch's *t* test.

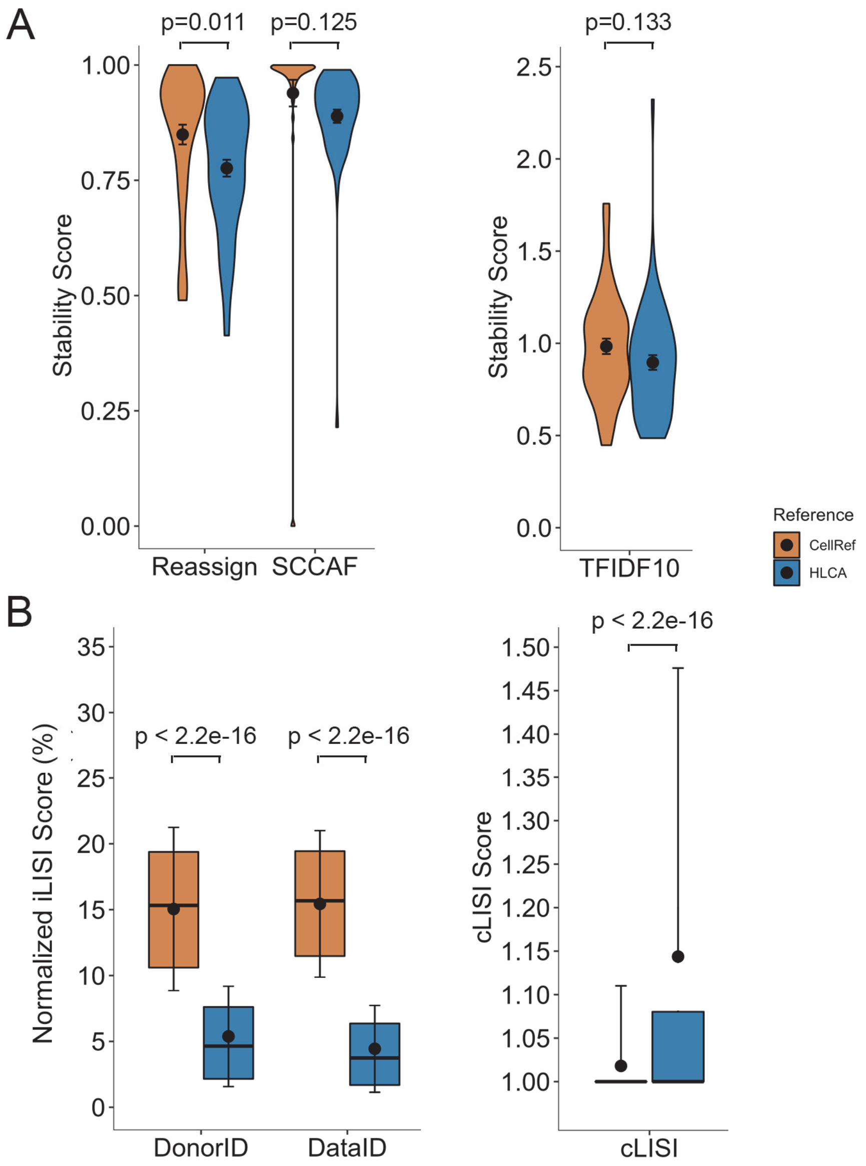

Supplementary Figure 12. Assessment of cell type stability in the LungMAP Mouse Lung CellRef. (A) We calculated the cell type stability metrics (Reassign, SCCAF[5], and TFIDF10) for each of the 40 cell types in mouse lung CellRef using scTriangulate[4] with default parameters. Distributions of stability scores are visualized using violin plots. The black dots and error bars represent mean±SEM. (**B**) Evaluation of cell type accuracy in mouse lung CellRef based on the expression of cell type marker genes. Accuracy was measured using the area under the receiver operator characteristics curve (AUC). Left: violin plot visualization of the distribution of the AUC values of the 40 cell types in mouse lung CellRef. The black dot and error bars represent mean±SEM. Mean AUC value is 95.8%. Right: An example of the ROC curve. Orange text showed the AUC value with (90% confidence interval).

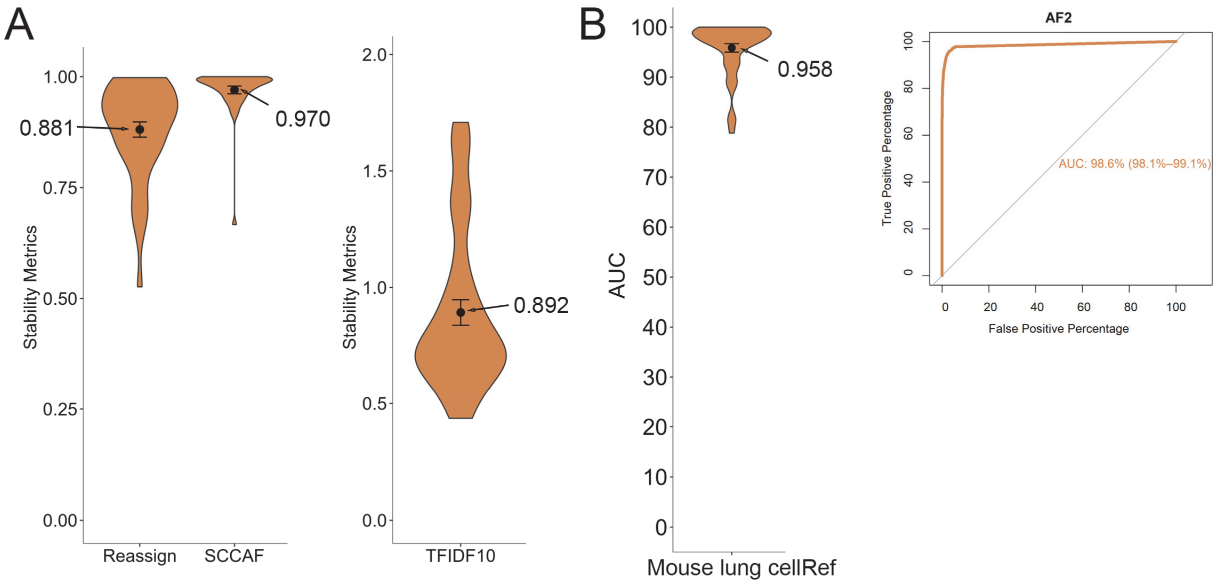

Supplementary References

1 Haghverdi, L., Lun, A. T. L., Morgan, M. D. & Marioni, J. C. Batch effects in single-cell RNA-sequencing data are corrected by matching mutual nearest neighbors. *Nat Biotechnol* **36**, 421-427, doi:10.1038/nbt.4091 (2018).

2 Korsunsky, I. *et al.* Fast, sensitive and accurate integration of single-cell data with Harmony. *Nat Methods* **16**, 1289-1296, doi:10.1038/s41592-019-0619-0 (2019).

3 Hao, Y. *et al.* Integrated analysis of multimodal single-cell data. *Cell* **184**, 3573-3587 e3529, doi:10.1016/j.cell.2021.04.048 (2021).

4 Li, G. *et al.* Decision level integration of unimodal and multimodal single cell data with scTriangulate. *Nat Commun* **14**, 406, doi:10.1038/s41467-023-36016-y (2023).

5 Miao, Z. *et al.* Putative cell type discovery from single-cell gene expression data. *Nat Methods*, doi:10.1038/s41592-020-0825-9 (2020).

6 Traag, V. A., Waltman, L. & van Eck, N. J. From Louvain to Leiden: guaranteeing well-connected communities. *Sci Rep* **9**, 5233, doi:10.1038/s41598-019-41695-z (2019).
