# Supplementary material for: Guided construction of single cell reference for human and mouse lung
Source: Nat Commun. 2023 Jul 29;14:4566. doi: 10.1038/s41467-023-40173-5 (PMC10387117; doi:10.1038/s41467-023-40173-5)
Supplement: Supplementary file 2 — Description of additional supplementary files [file 41467_2023_40173_MOESM2_ESM.pdf]

Description of additional supplementary files

Supplementary Data 1. Collection of single cell/single nucleus RNA-seq of human lung.

Supplementary Data 2. Cell type dictionary for the LungMAP Human Lung CellRef construction. p: positive cell type marker. n: negative cell type marker.

Supplementary Data 3. Sample information of Drop-seq of Mouse Lungs.

Supplementary Data 4. Cell type dictionary for the LungMAP Mouse Lung Development CellRef construction. p: positive cell type marker. n: negative cell type marker.

Supplementary Data 5. Cell type selective markers predicted by LungMAP Human Lung CellRef. Please see method for cell selective marker prediction.

Supplementary Data 6. Cell type selective markers predicted by LungMAP Mouse Lung CellRef. Please see method for cell selective marker prediction.
