# Supplementary material for: Guided construction of single cell reference for human and mouse lung
Source: Nat Commun. 2023 Jul 29;14:4566. doi: 10.1038/s41467-023-40173-5 (PMC10387117; doi:10.1038/s41467-023-40173-5)
Supplement: Supplementary file 9 — Reporting Summary [file 41467_2023_40173_MOESM9_ESM.pdf]

nature portfolio

Corresponding author(s): Yan Xu; Minzhe Guo

Last updated by author(s): Jun 17, 2023

Reporting Summary

Nature Portfolio wishes to improve the reproducibility of the work that we publish. This form provides structure for consistency and transparency in reporting. For further information on Nature Portfolio policies, see our Editorial Policies and the Editorial Policy Checklist.

Statistics

For all statistical analyses, confirm that the following items are present in the figure legend, table legend, main text, or Methods section.

n/a	Confirmed	
☐	☒	The exact sample size (*n*) for each experimental group/condition, given as a discrete number and unit of measurement
☐	☒	A statement on whether measurements were taken from distinct samples or whether the same sample was measured repeatedly
☐	☒	The statistical test(s) used AND whether they are one- or two-sided<br>*Only common tests should be described solely by name; describe more complex techniques in the Methods section.*
☒	☐	A description of all covariates tested
☐	☒	A description of any assumptions or corrections, such as tests of normality and adjustment for multiple comparisons
☐	☒	A full description of the statistical parameters including central tendency (e.g. means) or other basic estimates (e.g. regression coefficient) AND variation (e.g. standard deviation) or associated estimates of uncertainty (e.g. confidence intervals)
☐	☒	For null hypothesis testing, the test statistic (e.g. *F*, *t*, *r*) with confidence intervals, effect sizes, degrees of freedom and *P* value noted<br>*Give P values as exact values whenever suitable.*
☒	☐	For Bayesian analysis, information on the choice of priors and Markov chain Monte Carlo settings
☒	☐	For hierarchical and complex designs, identification of the appropriate level for tests and full reporting of outcomes
☐	☒	Estimates of effect sizes (e.g. Cohen's *d*, Pearson's *r*), indicating how they were calculated

Our web collection on statistics for biologists contains articles on many of the points above.

Software and code

Policy information about availability of computer code

Data collection	10X Cell Ranger (v3 and v5) was used to perform sequencing read alignment and UMI gene expression matrix generation using 10X single cell RNA-seq data. Drop-seq tool (https://github.com/broadinstitute/Drop-seq/, version 2.3.0) was used to align and quantify Drop-seq data.
Data analysis	Code from the present study can be found at https://github.com/xu-lab/CellRef. The CellRef pipeline utilized several open-source and previously published R packages and pipelines, including R (v4.1.0), Monocle 3 (v1.0.0), Seurat (v4.1.0), SingleR (v1.6.1), Harmony (v0.1.0), RobustRankAggreg (v1.2.1), Scrublet (v0.2.3), DropletUtils (v1.4.3), SoupX (v1.6.2), gprofiler2 (v0.2.1). R packages BayesianOptimization (v1.2.0), pROC (v1.18.0), pheatmap (v1.0.12), LISI (v1.0) and python pacakge scTriangulate (v0.12.0) were used in the evaluation analysis. Please see detailed description in Methods section of the manuscript and access the code in the github (https://github.com/xu-lab/CellRef).

For manuscripts utilizing custom algorithms or software that are central to the research but not yet described in published literature, software must be made available to editors and reviewers. We strongly encourage code deposition in a community repository (e.g. GitHub). See the Nature Portfolio guidelines for submitting code & software for further information.

Data

Policy information about availability of data

All manuscripts must include a data availability statement. This statement should provide the following information, where applicable:

- Accession codes, unique identifiers, or web links for publicly available datasets
- A description of any restrictions on data availability
- For clinical datasets or third party data, please ensure that the statement adheres to our policy

Published single cell/nucleus RNA-seq of human lung used in the human lung CellRef are available in the Gene Expression Omnibus under accession codes GSE135893 [https://www.ncbi.nlm.nih.gov/geo/query/acc.cgi?acc=GSE135893], GSE136831 [https://www.ncbi.nlm.nih.gov/geo/query/acc.cgi?acc=GSE136831], GSE122960 [https://www.ncbi.nlm.nih.gov/geo/query/acc.cgi?acc=GSE122960], GSE134174 [https://www.ncbi.nlm.nih.gov/geo/query/acc.cgi?acc=GSE134174] GSE161382 [https://www.ncbi.nlm.nih.gov/geo/query/acc.cgi?acc=GSE161382], GSE171524 [https://www.ncbi.nlm.nih.gov/geo/query/acc.cgi?acc=GSE171524], in the European Genome-phenome Archive under accession code EGAS00001004082 [https://ega-archive.org/studies/EGAS00001004082], and in the Synapse.org under accession code syn21041850 [https://www.synapse.org/#!Synapse:syn21041850]. The LungMAP CCHMC and UPenn data used in this study are available in the LungMAP.net under accession code LMEX0000004396 [https://lungmap.net/breath-omics-experiment-page/?experiment_id=LMEX0000004396].

Drop-seq of mouse lung data used in the mouse lung CellRef are available in the Gene Expression Omnibus under accession code GSE122332 [https://www.ncbi.nlm.nih.gov/geo/query/acc.cgi?acc=GSE122332] and in the LungMAP.net under accession codeLMEX0000004397 [https://lungmap.net/breath-omics-experiment-page/?experiment_id=LMEX0000004397].

Published single cell RNA-seq of human lung data used in the evaluation analysis are available in the Gene Expression Omnibus under accession codes GSE178362 [https://www.ncbi.nlm.nih.gov/geo/query/acc.cgi?acc=GSE178362], GSE135893 [https://www.ncbi.nlm.nih.gov/geo/query/acc.cgi?acc=GSE135893], GSE135851 [https://www.ncbi.nlm.nih.gov/geo/query/acc.cgi?acc=GSE135851], and GSE149563 [https://www.ncbi.nlm.nih.gov/geo/query/acc.cgi?acc=GSE149563].

The HLCA core reference (version 1.0) used in the benchmarking analysis is available at FASTGenomics under accession code dataset-427f1eee6dd44f50bae1ab13f0f3c6a9 [https://beta.fastgenomics.org/datasets/detail-dataset-427f1eee6dd44f50bae1ab13f0f3c6a9].

Web interfaces for the human and mouse lung CellRefs are available at Lung Gene Expression Analysis (LGEA) web portal (https://research.cchmc.org/pbge/lunggens/CellRef/LungMapCellRef.html) and LungMAP.net (https://lungmap.net/cell-cards/, "CellRef scRNA-seq" tab).

Human research participants

Policy information about studies involving human research participants and Sex and Gender in Research.

Reporting on sex and gender	Supplementary Data 1 listed sex of all datasets used in this study.
Population characteristics	This collection contains data from similar numbers of female and male donors (n=48 and 55, respectively; 1 unannotated). Population characteristics (sex and age) are outlined in Figure 1 and Supplementary Data 1.
Recruitment	Data was obtained from published and unpublished research. We collected 10 large-scale sc/snRNA-seq datasets (8 published and 2 unpublished) from the four regions of human lung: Vanderbilt_cohort (n=10 donors; parenchyma), Northwestern_cohort (n=8 donors; parenchyma), Yale_cohort (n=28 donors; parenchyma), CNSR_cohort (n=9 donors; trachea/bronchi/parenchyma), Stanford_cohort (n=3 donors; bronchi/parenchyma), NJH_cohort (n=15 donors; trachea), CCHMC_LungMAP_cohort (n=5, bronchus SMG), UCSD_LungMAP_cohort (n=3, small airway), Columbia_COVID19_cohort (n=7, parenchyma), and UPenn_LungMAP_cohort (n=16, parenchyma). The unpublished data are available from LungMAP consortium web portal (https://lungmap.net/explore-data/).
Ethics oversight	The present study does not meet the NIH definition of human subject research, but all institutional procedures required for human subject research were followed throughout the reported experiments. De-identified human tissue samples were obtained from human lung transplant donors under institutional IRB approval by the University of Pennsylvania and University of North Carolina at Chapel Hill Institutional Review Board.

Note that full information on the approval of the study protocol must also be provided in the manuscript.

Field-specific reporting

Please select the one below that is the best fit for your research. If you are not sure, read the appropriate sections before making your selection.

☒ Life sciences ☐ Behavioural & social sciences ☐ Ecological, evolutionary & environmental sciences

For a reference copy of the document with all sections, see nature.com/documents/nr-reporting-summary-flat.pdf

Life sciences study design

All studies must disclose on these points even when the disclosure is negative.

Sample size	In total, 505,256 lung cells from 148 sc/snRNA-seq of normal human lung samples from 104 donors were used for the study. In addition, we performed a power analysis to calculate the minimum number of cells required for a human lung cell type ($\alpha = 0.01$, $\beta = 0.2$, two-tailed t test). The mouse lung CellRef was constructed using Drop-seq (n=95,658 cells) from 17 mouse lung samples from eight developmental time points.
Data exclusions	Pre-filtering was applied to remove cells with low quality as described in the Methods section of the manuscript.
Replication	The human lung CellRef was constructed using single cell RNA-seq data from 104 donor lungs from 10 datasets generated by different research teams/centers. Additionally, single cell RNA-seq of human lung (27 biological replicates from 5 normal and 14 diseased lungs) were used to evaluate the performance. The mouse lung CellRef was constructed using Drop-seq of mouse lung from eight developmental time points. Two to three replicates per time point. All replication attempts were successful.
Randomization	Human lung CellRef collected 10 data cohorts at their completeness.<br>Mice utilized for Drop-seq experiments were randomly selected and grouped by age.
Blinding	The analysts were not involved in any of the data generation. Cell clustering was performed using an unsupervised clustering method therefore the initial cell type mapping was unbiased (can be interpreted as blinded design).

Reporting for specific materials, systems and methods

We require information from authors about some types of materials, experimental systems and methods used in many studies. Here, indicate whether each material, system or method listed is relevant to your study. If you are not sure if a list item applies to your research, read the appropriate section before selecting a response.

Materials & experimental systems

n/a	Involved in the study	
☒	☐	Antibodies
☒	☐	Eukaryotic cell lines
☒	☐	Palaeontology and archaeology
☐	☒	Animals and other organisms
☒	☐	Clinical data
☒	☐	Dual use research of concern

Methods

n/a	Involved in the study	
☒	☐	ChIP-seq
☒	☐	Flow cytometry
☒	☐	MRI-based neuroimaging

Animals and other research organisms

Policy information about studies involving animals; ARRIVE guidelines recommended for reporting animal research, and Sex and Gender in Research

Laboratory animals	C57BL/6J mice (Jackson Laboratories), embryonic days (E) 16.5, 18.5 to postnatal days (PND) 1, 3, 7, 10, 14, 28, were used for single cell RNA-seq experiments using Drop-seq. The animal care and use program at the CCHMC animal core fully complies with the Guide for the Care and Use of Laboratory Animals.
Wild animals	This study did not involve wild animals.
Reporting on sex	Supplementary Data 3 listed sex of all mouse lung Drop-seq datasets
Field-collected samples	This study did not involve filed-collected samples.
Ethics oversight	Animal protocols were approved by the Institutional Animal Care and Use Committee of Cincinnati Children's Hospital Medical Center.

Note that full information on the approval of the study protocol must also be provided in the manuscript.
